# Engineering of Surface Display Systems in *E. coli* for Recombinant Protein Production

**DOI:** 10.3390/biotech15040078

**Published:** 2026-09-15

**Authors:** Olga S. Kostareva, Polina I. Blinova, Svetlana V. Tishchenko, Ilya A. Kolyadenko, Anatoly S. Glukhov, Irina A. Eliseeva, Alisa O. Mikhaylina

**Affiliations:** Institute of Protein Research, Russian Academy of Sciences, 142290 Pushchino, Russiasveta@vega.protres.ru (S.V.T.);

**Keywords:** *E. coli* autotransporter protein, protein export optimization, anchor protein, SNAC cleavage

## Abstract

Isolation of various groups of protein preparations is a crucial task in many areas of biochemical research. Creating an effective, simple and economically feasible system for recombinant protein production is highly valuable for both basic research and applied biotechnology. Most laboratory studies focus on bacterial systems for heterologous gene expression. However, such systems can encounter major difficulties with target proteins that have specific properties. Some proteins can aggregate, form insoluble inclusion bodies or undergo proteolytic cleavage during overproduction. Moreover, they can be toxic, and thus adversely affect the host cell when overproduced. These issues can be circumvented by using cellular secretion systems to export the target protein from the cell during production. The present study compares three established systems for exporting target proteins to the bacterial cell surface. Three model proteins were selected: the cytokine IL-17A, the catalytic domain of TEV protease, and the calcium-binding domain of nucleobindin 1. Three protein export systems were used: the first was based on the *E. coli* outer membrane OmpA protein, the second on the *E. coli* Ag43 autotransporter of the AIDA-I family, and the third on the INP of *Pseudomonas syringae*. Recombinant proteins were cleaved by site-directed chemical proteolysis.

## 1. Introduction

*Escherichia coli* is one of the best-studied and most widely used expression system for producing heterologous recombinant proteins in both basic research and the biotechnology industry. However, the production of some complex proteins faces significant limitations. For example, some proteins form insoluble inclusion bodies when overproduced, requiring denaturation and subsequent renaturation. In addition, some overproduced peptides can be susceptible to proteolytic cleavage or can themselves adversely affect essential processes in the host strain. Possible solutions include periplasmic expression, protein export into the culture medium, or protein display on the cell surface. Periplasmic expression presents a challenge for releasing periplasmically expressed proteins at scale: only the outer membrane of the bacterial cell must be disrupted to release the target protein from the periplasmic space without affecting the cytoplasmic membrane. A cell-surface protein display system could overcome this difficulty. Like periplasmic expression, cell-surface display largely avoids disadvantages such as intracellular aggregation, proteolytic cleavage of the target protein, and the accumulation of improperly folded protein molecules. For toxic proteins, secretion also prevents the product from affecting essential processes in the expression host. Moreover, excretion of the target protein from the cell simplifies protein purification.

The first successful system for displaying full-length heterologous proteins on the surface of *E. coli* cells was Lpp-OmpA. The transmembrane domains of β-barrel-forming *E. coli* outer membrane proteins (OmpA, OmpT, OmpX, OmpC, etc.) [1,2,3,4] are often used as carrier proteins to facilitate the translocation of target proteins to the cell surface. Some systems also utilize the *E. coli* lipoprotein Lpp to anchor the chimeric construct in the outer membrane [1,2].

Monomeric autotransporters (ATs) belonging to the AIDA-I family are also widely used to expose the recombinant proteins on *E. coli*’s surface. The coding sequence for the extracellular domain AT (N-terminus) is replaced with a recombinant protein domain. Three AT proteins from the *E. coli* AIDA-I family are used for recombinant protein production: AIDA-I from enteropathogenic *E. coli* O126: H27 [5,6,7,8,9,10] and the Ag43 [11,12,13,14,15,16] and YfaL [17] aggregation factors from non-pathogenic *E. coli* MG1655. This system is widely used to display proteins such as β-lactamase, cellulase, scFv antibodies, cyclodextrine glucane transferase, cellulose-binding domain and chitin-binding domain on the surface of *E. coli* cells [1,18,19].

Another widely used system for displaying target proteins on the surface of *E. coli* is based on ice nucleation protein (INP). INP is a family of outer membrane proteins found in several phytopathogenic bacteria: InaK, InaQ and InaV from *P. syringae*; InaA from *Erwinia ananas*; InaX from *Xanthomonas campestris*; and InaPb from *Pseudomonas borealis.* Both full-length INP constructs [20,21,22] and its truncated forms [22,23,24,25,26,27,28,29,30,31,32,33,34,35,36,37,38,39] have been used to display functionally active enzymes on the surface of *E. coli* cells. The target protein is located at the C-terminus of the chimeric protein. INP offers several advantages as a system for exporting and displaying recombinant proteins: (1) it imposes no apparent limitation on the size of the exported protein; (2) it can export proteins possessing cofactors or disulfide bonds; (3) INPs can be present on the cell surface of *E. coli* in large amounts without affecting cell viability unlike the endogenous membrane protein OmpA [5]. INPs are often used to create whole-cell biocatalysts displaying, for example, 119 kDa cytochrome P450 BM3 [29], 77 kDa NADPH-cytochrome P450 oxidoreductase [24], and alditol oxidase [40]. All these proteins possess cofactors, indicating that INP can export fully folded proteins with their cofactors already incorporated. INP has also been used to display organophosphate hydrolases [28], xylose dehydrogenase [35] and carbonic anhydrases (CAs) [40].

The most widely used systems for exporting and displaying recombinant proteins in *E. coli* and their applications were comprehensively described in a recent review [41].

Systems that export target proteins to the cell surface are widely used for industrial and biotechnological purposes, for example, to create carrier cells (particularly heavy metal adsorbers) [42] and in laboratory diagnostics [6,7]. However, they are relatively rarely applied in the area of recombinant protein production. One challenge is the need for a cleavage agent to separate the target protein from the carrier protein. Specific proteases are often used to cleave the sequence linking the target and carrier proteins, but proteases can also act nonspecifically. Their use may require additional processing, and the target protein preparation must be purified to remove protease contaminants. This requirement, among others, limits the application of surface display systems for recombinant protein production. A technique for the specific nickel-ion-mediated cleavage of a protein sequence (-GSHHW-, the SNAC-tag) was recently described [43]. With this approach, an additional step to remove the cleavage agent from the target protein preparation is not required.

Our current work presents basic laboratory-scale research that combines several systems for exporting a target protein to the cell surface with the site-specific chemical proteolysis. As a result, novel modified systems were constructed in which the secreted target protein, anchored to the cell surface, was released by chemical proteolysis. Both monomeric target proteins (the catalytic domain of TEV protease and the calcium-binding domain of nucleobindin 1) and a dimeric protein stabilized by disulfide bonds (IL-17A) were selected.

## 2. Materials and Methods

### 2.1. Design of Genetic Constructs

#### 2.1.1. Lpp_OmpA_SNAC

The expression platform for the Lpp_OmpA export system was created using a modified pET-11a vector under control of T7 promoter. This platform contains sequences encoding the following components: the signal peptide and first nine amino acids of mature *E. coli* Lpp protein; part of the transmembrane domain of *E. coli* OmpA protein; and SNAC-tag (-GSHHW-) (see the Supplementary File for details). Oligodeoxynucleotide primers were synthesized according to the nucleotide sequences encoding the corresponding regions of Lpp and OmpA (P1 and P2; P5 and P6; Supplementary Table S1) (Evrogen and Syntol, Moscow, Russia). Primers P2 and P3 contained overlapping sequences for amplification of the chimeric Lpp_OmpA_SNAC gene. P4 primer contained a sequence encoding the SNAC-tag. In the first stage, the Lpp and OmpA genes were amplified using primer pairs P1–P2 and P3–P4, respectively, with genomic DNA from *E. coli* (strain K-12 substr. MG1655). In the second stage, the chimeric Lpp-OmpA_SNAC construct was amplified using the P1–P4 primer pair and the PCR products of Lpp and OmpA genes as a template.

Platform constructs based on pET-11a and containing the chimeric Lpp_OmpA_SNAC gene were obtained by in vivo recA-independent recombination (IVA cloning), as described previously [44]. For this purpose, pET-11a vector was amplified using P5 and P6 primers (Supplementary Table S1) (Evrogen and Syntol, Russia). The PCR fragment encoding the chimeric Lpp_OmpA_SNAC gene was amplified using the P7–P8 primer pair (Evrogen and Syntol, Russia), which encoded regions complementary to the ends of the amplified pET-11a vector (Supplementary Table S1). The amplified pET-11a vector was treated with methylation-sensitive restriction endonuclease DpnI (Thermo Scientific, Waltham, MA, USA). The vector and chimeric gene construct were then mixed and transformed into competent *E. coli* XL1 (blue) cells. Transformants were grown on LB agar plates containing ampicillin as a selection marker (100 µg/mL).

Vectors harboring the genes of target proteins were also constructed by IVA cloning using the Lpp_OmpA platform. Primers were designed to amplify the genes encoding TEV protease, IL-17A and the CaBD protein of NUCB1 (P9–P14; Supplementary Table S1) (Evrogen and Syntol, Russia).

In the first stage, PCR fragments encoding TEV protease, IL-17A and CaBD were amplified using P9–P10, P11–P12, and P13–P14 primer pairs, respectively, and previously obtained genetic constructs carrying the corresponding genes. All constructs encoded an N-terminal 6xHis-tag, which was included for further studies of the target proteins. In the second stage, pET-11a_Lpp_OmpA_SNAC vector encoding the export system was amplified using the P4–P5 primer pair. PCR fragments of the TEV protease, IL-17A and CaBD genes were amplified using additional primer pairs P15–P16, P17–P18, and P19–P20, respectively, which carried regions homologous to the ends of the amplified pET-11a_Lpp_OmpA_SNAC vector (Supplementary Table S1). The amplified vector was digested with the methylation-sensitive restriction endonuclease DpnI (Thermo Scientific). The PCR fragments of the genes and vector were then mixed and introduced into competent *E. coli* XL1 (blue) cells by transformation.

Constructs containing a modified SNAC-tag with an additional N-terminal linker and an extended SNAC-tag sequence (gggsgggsYFLPGSHHWG) were obtained by site-directed mutagenesis [45]. The previously obtained plasmids (pET-11a_Lpp_OmpA_SNAC_TEV, pET-11a_Lpp_OmpA_SNAC_IL-17A, pET-11a_Lpp_OmpA_SNAC_CaBD) served as PCR templates. The vectors were amplified using P21–P22 primers, which contained the nucleotide sequences required to extend the SNAC-tag and linker as well as regions complementary to the parent plasmids.

All nucleotide sequences of the derived genetic constructs were confirmed by Sanger sequencing (Evrogen, Moscow, Russia).

#### 2.1.2. SNAC_Ag43

The expression platform based on Ag43 export system was created using a modified pET-11a vector under the control of the T7 promoter. The chimeric construct contains sequences encoding PelB signal peptide, SNAC-tag (-GSHHW-), and the translocation domain (700–1039 aa) of Ag43, an *E. coli* autotransporter belonging to the AIDA-I family (Supplementary File). Primers P23 and P24, and P25 and P26 were synthesized according to the nucleotide sequences of the PelB signal peptide and Ag43 translocation domain, respectively (Supplementary Table S1) (Evrogen and Syntol, Moscow, Russia). These primers encoded overlapping sequences and the SNAC-tag sequence for amplification of SP_PelB__SNAC_Ag43 chimeric construct. In the first stage, PCR fragments of SP PelB and Ag43 were amplified using P23–P24 and P25–P26 primer pairs, respectively, with genomic DNA from *E. coli* K-12 substr. MG1655 as the template. In the second stage, the chimeric SP_PelB__SNAC_Ag43 sequence was amplified using the P23–P26 primer pair and the first-stage PCR products encoding SP PelB and Ag43 translocation domain as templates.

pET-11a construct containing the chimeric SP_PelB__SNAC_Ag43 gene was generated by IVA cloning as described above (Section 2.1.1) using P5 and P6 primers to amplify the pET-11a vector and the P27–P28 primer pair to amplify the chimeric SP_PelB__SNAC_Ag43 gene.

Vectors containing inserts encoding the target proteins were also obtained by IVA cloning using the Ag43 export system construct, as described above (Section 2.1.1). The vector pET-11a_SP_PelB__SNAC_Ag43 was amplified using the P23–P29 primer pair (Supplementary Table S1), whereas the previously obtained PCR fragments of the genes encoding TEV protease, IL-17A and CaBD (Section 2.1.1) were amplified using P30–P31, P32–P33, and P34–P35 primer pairs, respectively. These primers contained regions homologous to the ends of the amplified pET-11a_SPpelb_SNAC_Ag43 vector (Supplementary Table S1). The genes of the target proteins also encoded N-terminal 6His-tag.

#### 2.1.3. INP_SNAC

The expression platform based on the N-terminal domain of the INP from *P. syringae* was constructed in the pET-22b(+) vector under the control of the T7 promoter. The chimeric construct was composed of sequences encoding the N-terminal fragment of the INP (N-domain, 1–167 aa, and a part of CRD, 168–203 aa), a linker region (GGGSG), and the SNAC-tag sequence (-GSHHW-) (Supplementary File). The N-terminal PelB leader sequence required to export the recombinant protein to the periplasmic space of the host cell is encoded by pET-22B(+) vector. Primers P36 and P37 (Supplementary Table S1), which encoded the linker and SNAC-tag sequences, were used to amplify the N-terminal region of the INP (1–203 aa). Genomic DNA from *P. syringae* pv syringae (strain 3023) was used as the PCR template.

The platform construct based on pET-22b(+) and containing the INP_SNAC gene was generated by IVA cloning as described above (Section 2.1.1). For this purpose, the pET-22b(+) vector was amplified using the P38–P39 primer pair (Supplementary Table S1). The PCR fragment encoding the chimeric INP_SNAC gene was amplified using P40–P41 primers (Supplementary Table S1), which were partially homologous to the ends of the amplified pET-22b(+) vector.

Vectors encoding the target proteins were constructed by IVA cloning using the pET-22b_INP_SNAC plasmid. The pET-22b_INP_SNAC vector was amplified using P42–P43 primers (Supplementary Table S1), whereas previously obtained PCR fragments encoding TEV protease, IL-17A and CaBD were amplified using P44–P45, P46–P47, and P48–P49 primer pairs, respectively. These primers were partially homologous to the ends of the pET-22b_INP_SNAC vector (Supplementary Table S1). The target protein genes also encoded an N-terminal 6His-tag.

### 2.2. Expression of Platform Constructs and Constructs with Target Proteins

The T7 expression system was used to create strains for protein overproduction. Since the genes encoding IL-17A, TEV protease and CaBD possess codons that are rare in *E. coli*, the Rosetta strain was used to analyze the expression of the chimeric genes. Rosetta(DE3) cells were transformed with the corresponding plasmids and grown overnight on solid LB medium supplied with antibiotics (100 μg/mL of ampicillin, 30 μg/mL of chloramphenicol) at 37 °C. Several colonies were inoculated into liquid LB medium supplied with antibiotics and grown overnight at 37 °C and 160 rpm. 50 mL of liquid LB medium supplemented with antibiotics was inoculated with an overnight culture at a 1:100 ratio (*v*/*v*). The culture was grown at 37 °C and 160 rpm up to OD_600_ ≈ 0.5. The cells were then supplemented with 0.5 mM of IPTG and incubated under the same conditions for 3 h before being harvested by centrifugation.

Each experiment included at least three biological replicates. The level of recombinant protein production was analyzed by gel densitometry following SDS-PAGE using Image J software (the Windows version of ImageJ bundled with Java 8). Means and maximum deviations were calculated using Microsoft Office 16 Excel.

### 2.3. Effect on Cell Culture Growth (Optical Density)

To estimate the effect of three derived platforms (Lpp_OmpA_SNAC, SNAC_Ag43, INP_SNAC) and the constructs displaying the target proteins (CaBD, IL-17A and TEV protease) on *E. coli* cell growth, culture optical density (OD_600_) was compared 3 h after inducer addition. Untransformed *E. coli* Rosetta(DE3) cells and *E. coli* Rosetta(DE3) cells transformed with pET-28a vectors harboring CaBD or IL-17A or pET-21a_TEV lacking an export system (vectors for cytoplasmic expression) served as controls [46,47]. Each experiment included three biological replicates.

The statistical estimation of obtained results was then performed. Standard deviation was obtained from at least three independent experiments. *p*-value was calculated using Microsoft Office 16 Excel T.TEST function. Means of two datasets (OD_600_ after 3 h after IPTG addition for: Rosetta(DE3) cells and Rosetta(DE3) cells overproducing different anchors; Rosetta(DE3) cells overproducing cytoplasmic target proteins and Rosetta(DE3) cells with surface-displayed target proteins) were compared and assessed if their difference is statistically significant.

### 2.4. Fluorescent Microscopy

*E. coli* Rosetta(DE3) cells (1 optical unit at 600 nm) expressing cytoplasmic CaBD, CaBD in one of the surface display systems (CaBD_SNAC_Ag43 or Lpp-OmpA_SNAC_CaBD) or only anchoring proteins of one of the display systems (SNAC_Ag43 or Lpp-OmpA_SNAC) were harvested by centrifugation and washed three times with PBS. Then, 100 μL of the cells resuspended in PBS were immobilized on glass slides pretreated with Poly-L-lysine (5 mg/mL) for 15 min. The slides were washed three times with PBS. Cells on poly-L-lysine-coated coverslips were fixed with 4% PFA in PBS (30 min, RT), washed three times with PBS, blocked with blocking solution (5% FBS in PBS-T) for 30 min at RT, and incubated with primary antibodies (rabbit serum against full-length NUCB1 protein (1:500)) in blocking solution overnight at 4 °C. After three washes with PBS-T, coverslips were incubated with secondary antibody (Alexa-488-conjugated anti-rabbit IgG, 1:200, A11008, Invitrogen, Carlsbad, CA, USA) and Hoechst 33342 (1 μg/mL, Paneco, Gorki Leninskiye Urban Settlement, Russia) in blocking solution for 1 h at RT. Samples were washed three times with PBS-T, mounted with Fluoromount (F4680, Sigma, St. Louis, MO, USA), and imaged on a Leica TCS SPE microscope (Leica, Wetzlar, Germany). All solutions were prepared using 5% serum in PBS, and the volume was 100 μL. After each incubation stage, the immobilized cells were washed with PBS three times. A similar protocol that omitted incubation with polyclonal rabbit serum against full-length NUCB1 protein was used to control for nonspecific sorption of the Alexa-488-conjugated secondary anti-rabbit antibodies.

### 2.5. Production of TEV Protease, IL-17A and CaBD via E. coli Cell-Surface Display Systems

#### 2.5.1. Chemical Proteolysis of the Cell-Surface-Displayed Target Proteins by Ni Ions

The cells of the following overproducing strains,

Rosetta(DE3)_pET-11a_Lpp_OmpA_SNAC_TEV;
Rosetta(DE3)_pET-11a_Lpp_OmpA_SNAC_IL-17A;
Rosetta(DE3)_pET-11a_Lpp_OmpA_SNAC_CaBD;
Rosetta(DE3)_pET-11a_Lpp_OmpA_SNAC(+)_TEV;
Rosetta(DE3)_pET-11a_Lpp_OmpA_SNAC(+)_IL-17A;
Rosetta(DE3)_pET-11a_Lpp_OmpA_SNAC(+)_CaBD;
Rosetta(DE3)_pET-11a_TEV_SNAC_Ag43;
Rosetta(DE3)_pET-11a_IL-17A _SNAC_Ag43;
Rosetta(DE3)_pET-11a_CaBD _SNAC_Ag43;
Rosetta(DE3)_pet-22b_INP_SNAC_TEV;
Rosetta(DE3)_pet-22b_INP_SNAC_IL-17A;
and Rosetta(DE3)_pet-22b_INP_SNAC_CaBD

were harvested by centrifugation (10 °C, 5 min, 4000× *g*) 3 h after inducer addition. The pellet was washed with a solution of 50 mM of Tris-HCl and 100 mM of NaCl and centrifuged again (10 °C, 5 min, 4000× *g*). The pellet was then treated with the solution containing 100 mM of CHES (pH of 8.4), 100 mM of Acetone Oxime, 100 mM of NaCl, and 1 mM of NiCl_2_ (20% of the initial cell-culture volume) at 18 °C, 25 °C, and 37 °C for 1, 3 and 18 h, according to the method described by Dang [43] with minor modifications. The highest cleavage efficiency was observed after incubation at 37 °C for 18 h. The pellet and supernatant were separated by low-speed centrifugation (10 °C, 5 min, 4000× *g*).

The levels of the chimeric protein, cleavage target protein and cleavage anchor were analyzed by gel densitometry following SDS-PAGE using Image J software. The cleavage efficiency for all proteins was calculated as the sum of the cleavage target protein and the anchor. The means and maximum deviations were calculated using Microsoft Excel.

#### 2.5.2. Isolation and Purification of Target Proteins

The supernatant obtained after chemical proteolysis was immobilized onto Ni-NTA Agarose in the solution containing 50 mM of Tris-HCl (pH of 8.0), 500 mM of NaCl, and 10 mM of imidazole. The column was then washed with the same buffer solution. The 6His-taggged target protein was eluted using 250 mM of imidazole in the buffer solution of otherwise identical composition.

To destabilize the noncovalent interactions between the target protein and the cell membrane, the pellet obtained after chemical proteolysis was suspended in the solution containing 50 mM of Tris-HCl (pH of 8.0), 200 mM of NaCl, and either 1 or 2 M of urea for 18 h at 4 °C. The pellet and supernatant were separated by centrifugation. The fraction containing the 6His-tagged target protein was then immobilized onto Ni-NTA Agarose in the solution containing 50 mM of Tris-HCl (pH of 8.0), 500 mM of NaCl, and 10 mM of imidazole. The target protein was eluted using 250 mM of imidazole in a buffer of otherwise identical composition. Concentrations of final preparations for all target protein have been estimated spectrophotometrically.

#### 2.5.3. Isolation of Chimeric Proteins Lpp_OmpA_SNAC_TEV and Lpp_OmpA_SNAC_IL-17A

Cell pellet from Rosetta(DE3)_pET-11a_Lpp_OmpA_SNAC_TEV and Rosetta(DE3)_pET-11a_Lpp_OmpA_SNAC_IL-17A cultures were resuspended in 50 mM of Tris-HCl (pH of 7.5) and 6 M of Gu-HCl. The cells were then disintegrated by ultrasonication at 4 °C. The lysate was immobilized onto Ni-NTA Agarose in the solution containing 50 mM of Tris-HCl (pH of 8.0), 200 mM of NaCl, 6M of urea, and 10 mM of imidazole. The resin was washed with the same solution, and the protein was eluted using 250 mM of imidazole in the buffer of otherwise identical composition. The chimeric protein preparation was dialyzed against the solution containing 2 M of urea, 100 mM of CHES (pH of 8.4), 100 mM of NaCl, 100 mM of Acetone Oxime, and 1 mM of NiCl_2_.

### 2.6. Analysis of the Specific Interaction of IL-17A with Antibody

The IL-17A preparations were dialyzed against the solution containing 10 mM of sodium acetate (pH of 5.0), 100 mM of NaCl, and 10 mM of imidazole.

Four microtubes, each containing 20 μL of Ni-NTA Agarose, were used. Three of them contained IL-17A immobilized onto Ni-NTA Agarose; the fourth served as a control for interaction between the IL-17A-binding antibody netakimab and Ni-NTA Agarose. Netakimab was added to one of tube containing immobilized IL-17A at an IL-17A:netakimab molar ratio = 1:1.2. Ribosomal protein L1 was added to a second tube containing immobilized IL-17A at an IL-17A:L1 molar ratio of 1:1.8. The Ni-NTA Agarose was then washed with a buffer solution containing 10 mM of imidazole. Elution was performed using a buffer containing 250 mM of imidazole. Both the eluate and flow-through were analyzed by electrophoresis in SDS-PAGE.

### 2.7. Estimation of Specific Proteolytic Activity of TEV Protease

To estimate the functional activity, two TEV protease preparations were used: (1) TEV protease obtained after the proteolysis and purification of the INP_SNAC_TEV protein and (2) TEV protease produced in recombinant bacteria without an export system—TEVc.

The solution of TEV protease was supplemented with the archaeal protein from the SmAP2 family possessing TEV protease cleavage site in the buffer solution composed of 50 mM of Tris-HCl (pH of 7.5), 100 mM of NaCl, 2 mM of EDTA, and 1 mM of DTT. The substrate was mixed with TEV protease at a 20:1 molar ratio, in accordance with the recommendations for commercial TEV protease preparations. The enzyme-substrate mixture was incubated at 4 °C for 16 h. The proteolytic efficiency was analyzed by electrophoresis in SDS-PAGE. The levels of the cleaved and non-cleaved substrates were assessed by gel densitometry following SDS-PAGE using Image J software; the data were then processed in Microsoft Excel.

### 2.8. Analysis of the Specific Interaction of CaBD with RNA Fragment

To estimate the RNA-binding activity of CaBD, two preparations were used: (1) CaBD obtained after the proteolysis and purification of Lpp-OmpA_SNAC(+)_CaBD protein and (2) CaBD produced in recombinant bacteria without an export system—CaBDc [46]. As a partner 5′-biotinylated chemically synthesized RNA oligomer (Syntol, Moscow, Russia) miR-200a-3p (5′-GUACCGAGCUCGAAUUUAACACUGUCUGGUAACGAUGU-3′) was used [46].

The miR-200a-3p was heated at 60 °C for 10 min and incubated for a further 10 min at 4 °C. miR-200a-3p was added to the tube containing CaBD obtained after the proteolysis of Lpp-OmpA_SNAC(+)_CaBD or CaBDc at an RNA:protein molar ratio of 1:0.5 or 1:1; the mixture was then incubated for 20 min at 22 °C. RNA–protein complexes were analyzed by electrophoresis using the Mini-PROTEAN II system (Bio-Rad, Hercules, CA, USA) on 12% gels under non-denaturing conditions in the buffer containing 90 mM of Tris-boric acid (pH of 8.2) at 100 V for 2.5 h. After electrophoresis, the gel was stained with ethidium bromide.

### 2.9. Isolation of the Recombinant TEV Protease and CaBD from Cytosolic Fraction of E. coli Cells

The cells of Rosetta(DE3) expression strain were transformed with pET-21_TEV vector harboring TEV protease gene without a cell-surface export tag. Expression was induced as described above.

The pellet of Rosetta(DE3)_pET-21_TEV cells was resuspended in 50 mM of Tris-HCl (pH of 7.5) and 500 mM of NaCl. The cells were then disintegrated by ultrasonication at 4 °C. The supernatant and cell debris were separated by centrifugation (10,000× *g*, 15 min). The lysate was immobilized onto Ni-NTA Agarose in the solution containing 50 mM of Tris-HCl (pH of 7.5), 200 mM of NaCl, and 10 mM of imidazole. The resin was washed with the same buffer solution. TEV protease was eluted using the same buffer with an increased imidazole concentration of 250 mM.

The CaBDc was obtained as previously described [46].

## 3. Results

Twelve genetic constructs were created using three export systems; each system was used to produce three target proteins.

### 3.1. Genetic Constructs Based on Lpp-OmpA Export System

**Figure 1:**
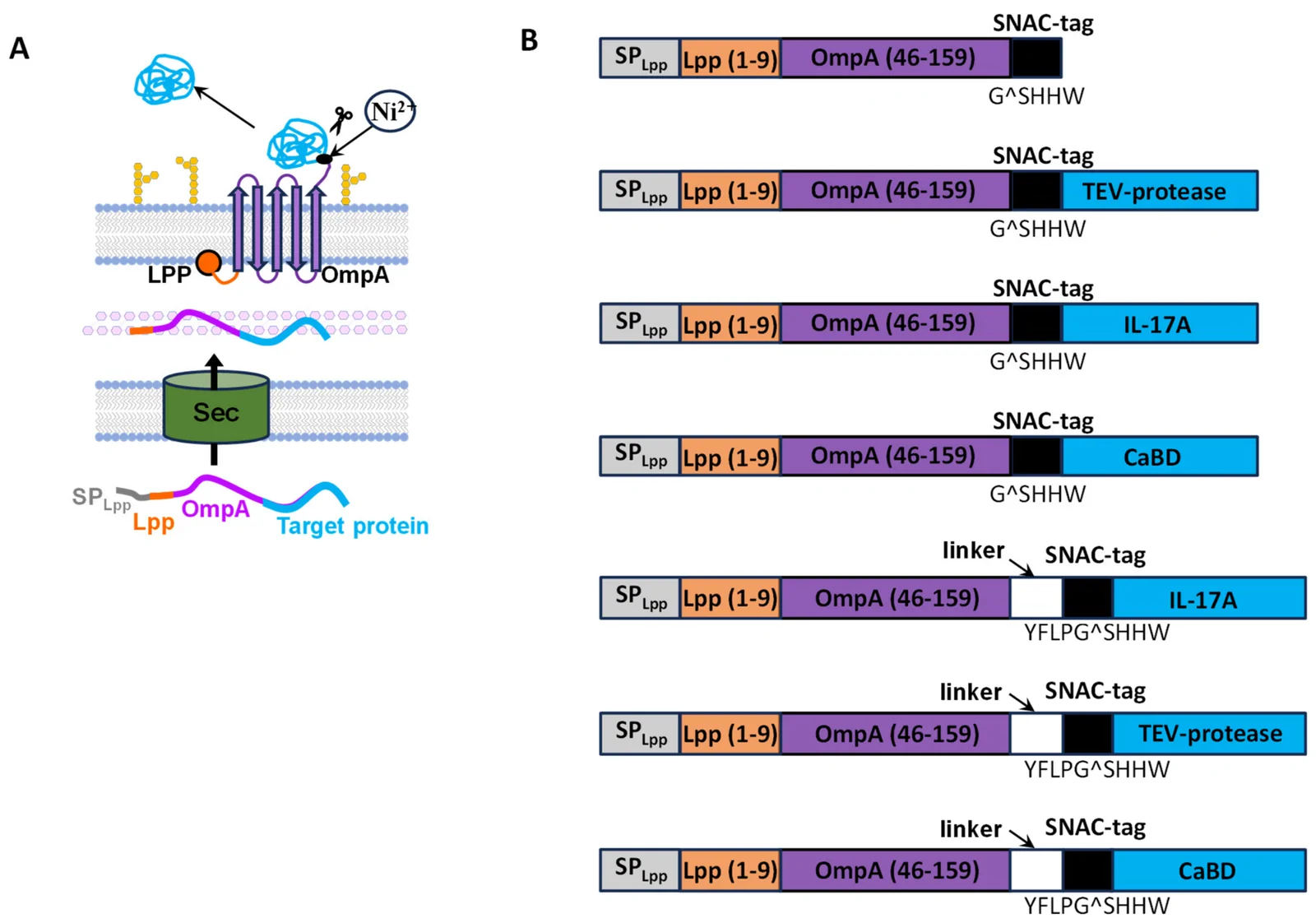
Schematic representation of the Lpp-OmpA export system created using the *E. coli* outer membrane protein OmpA. (**A**)—OmpA integration into the *E. coli* membrane and display of a recombinant protein. (**B**)—Design of constructs created using the Lpp-OmpA_SNAC and Lpp-OmpA_SNAC(+) export systems.

The Lpp_OmpA_SNAC platform construct, based on the outer membrane protein OmpA, contains a chimeric sequence encoding the signal peptide and the first nine amino acids of mature *E. coli* lipoprotein Lpp, an anchoring sequence (46–159 aa of mature OmpA protein) and a SNAC-tag, which permits nickel-ion-mediated chemical cleavage of the target protein at the GSHHW sequence. The Lpp signal peptide directs the chimeric protein to the periplasmic space, whereas the first nine amino acids of mature Lpp anchor it in the membrane. Five transmembrane segments of OmpA (46–159 aa of the mature OmpA protein) are required to display the target protein on the cell surface. The chimeric Lpp-OmpA construct (46–159 aa) has been shown to be the most effective for protein display on the surface of *E. coli* cells [1,48,49] (Figure 1A).

Using the Lpp-OmpA_SNAC export platform, genetic constructs harboring the genes encoding TEV protease, IL-17A, and the calcium-binding domain of NUCB1 (CaBD [46]) were created. To export the recombinant protein to the outer membrane of *E. coli*, the target protein sequence was located at the C-terminus of the chimeric Lpp-OmpA protein: Lpp-OmpA_SNAC_TEV, Lpp-OmpA_SNAC_IL-17A, and Lpp-OmpA_SNAC_CaBD (Figure 1B). The target protein sequences also encoded a 6His-tag.

The G^SHHWG sequence proved too short for effective nickel-ion-mediated cleavage of the target protein in the Lpp-OmpA_SNAC export system, as described below (Section 3.7.1). Constructs with the extended SNAC-tag, YFLPG^SHHWG, and added linker GGGSGGGS before extended SNAC-tag, were also produced using the Lpp-OmpA_SNAC platform (Lpp-OmpA_SNAC(+)) (Figure 1B and Supplementary File).

### 3.2. Genetic Constructs Based on Ag43 Autotransporter

**Figure 2:**
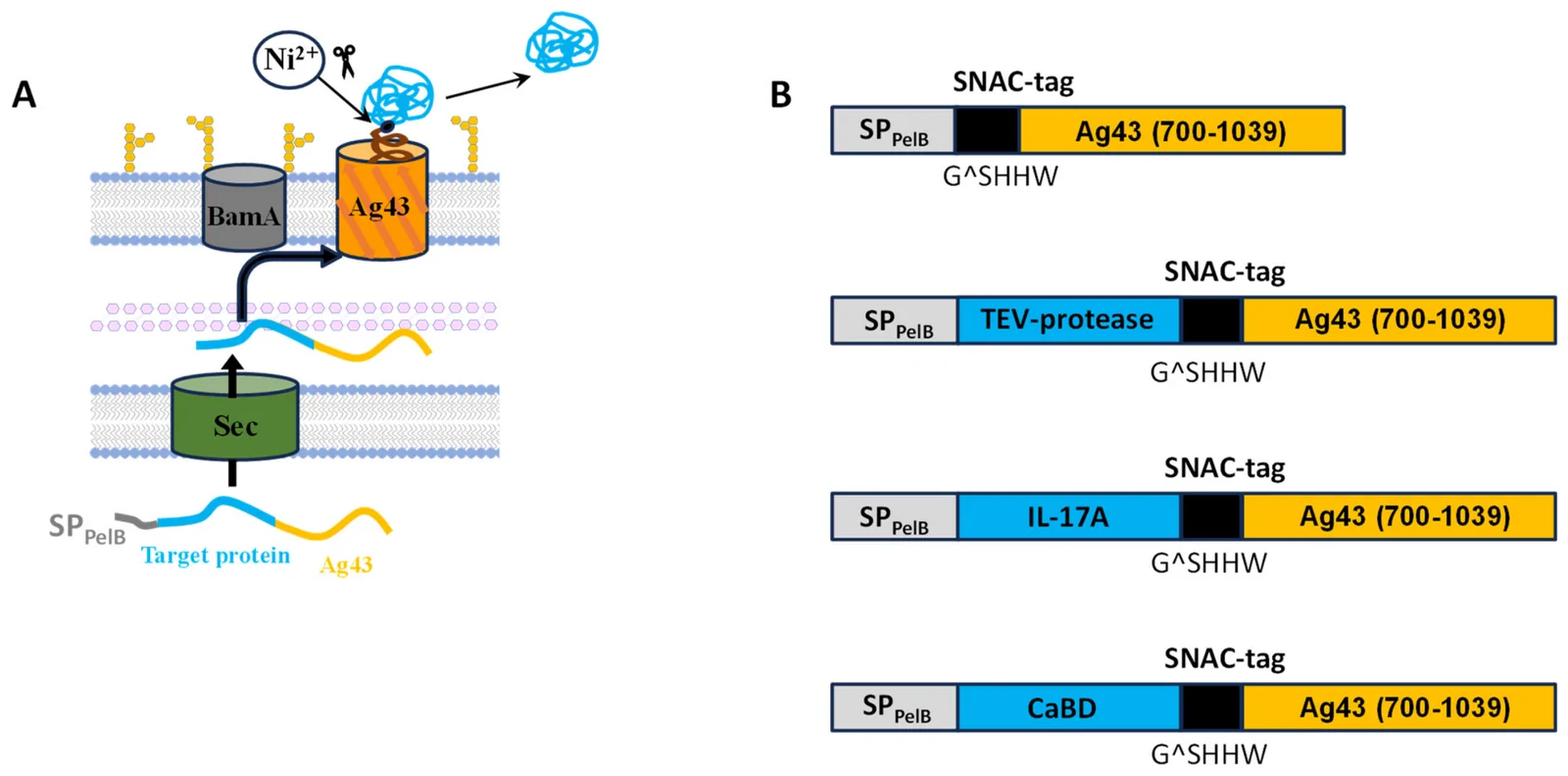
Schematic representation of the Ag43_SNAC export system created using the *E. coli*, an autotransporter of the AIDA-I family. (**A**)—Integration of the autotransporter into the *E. coli*’s outer membrane and display of the recombinant protein in the Ag43_SNAC export system. (**B**)—Design of constructs created using the Ag43_SNAC export system.

The SNAC_Ag43 platform construct was created using Ag43 protein, an autotransporter from the AIDA-I family. SNAC_Ag43 contains a chimeric sequence encoding the PelB signal peptide for export of the chimeric protein to the periplasmic space, a sequence for chemical cleavage of the target protein (SNAC-tag, GSHHW), and the Ag43 translocation domain (700–1039 aa) as an anchoring protein (Figure 2A). The autocatalytic cleavage site of the Ag43 autotransporter was removed to permit controlled cleavage of the target sequence by SNAC-tag- and nickel-ion-mediated chemical proteolysis.

Using the AIDA-I (Ag43) export platform construct, three genetic constructs encoding TEV protease, IL-17A and CaBD were created. In this system, the target proteins were located at the N-terminus of the encoded chimeric protein: TEV_SNAC_Ag43, IL-17A_SNAC_Ag43, and CaBD_SNAC_Ag43 (Figure 2B). The target protein sequences also encoded a 6His-tag.

### 3.3. Genetic Constructs Based on INP

**Figure 3:**
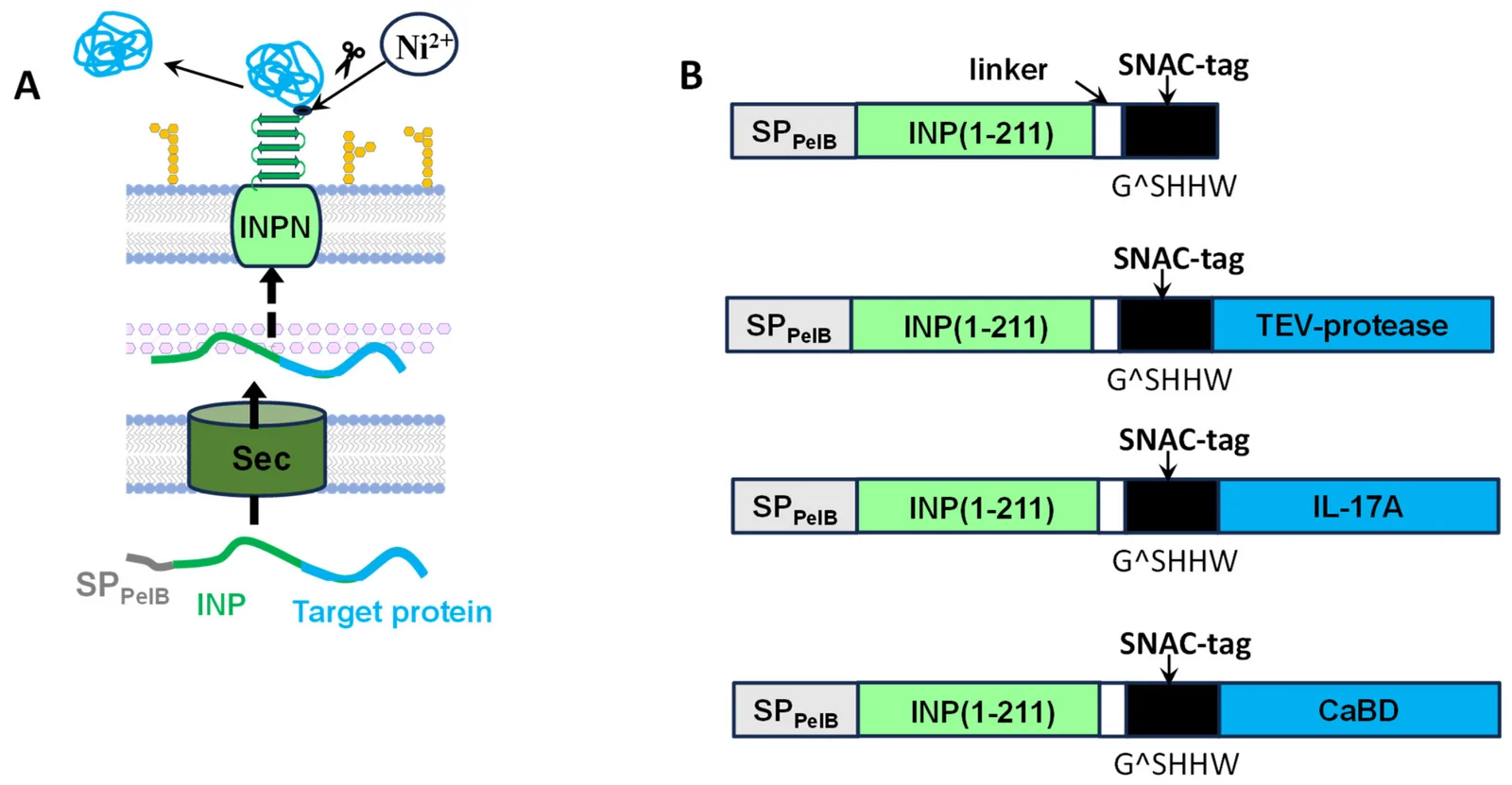
Schematic representation of the INP_SNAC export system created using the *P. syringae* INP. (**A**)—Integration of INP into the *E. coli*’s outer membrane and display of the recombinant target protein in the INP_SNAC export system. (**B**)—Design of constructs created using the INP_SNAC export system.

The INP_SNAC export platform construct was designed using the N-terminal domain of the INP from *P. syringae*. INPs are composed of three domains. The hydrophobic N-terminal domain is anchored to the outer membrane, and it directs the protein to the cell surface. The central repeated domain (CRD) consists of several turns that serve as templates for ice-crystal formation. The C-terminal domain is hydrophilic and is exposed to the extracellular space. The fragment of INP was chosen for the export platform due to the data presented by Zhu et al. in 2022 [39], who used this scaffold to display carbonic anhydrase. The INP_SNAC construct contains a chimeric sequence encoding the PelB signal peptide, an fragment of INP (1–203 aa) containing its N-terminal domain and part of the CRD as an anchoring protein, a linker region (GGGSGG), and a SNAC-tag sequence (GSHHW) for chemical cleavage of the target protein (Figure 3A).

Using the INP export platform construct, three genetic constructs encoding TEV protease, IL-17A and CaBD were created. To export the recombinant proteins to the outer membrane of *E. coli*, the target protein sequence was located at the C-terminus of the chimeric sequence in INP export systems: INP_SNAC_TEV, INP_SNAC_IL-17A, INP_SNAC_CaBD (Figure 3B).

### 3.4. Expression of Recombinant Protein Genes in Different Export Systems

First, expression was analyzed for all platform constructs without target proteins. All anchoring constructs were effectively overproduced in *E. coli* strain Rosetta(DE3) (Figure S1).

Chimeric proteins containing TEV, IL-17A, or CaBD were effectively produced in Rosetta(DE3) using all three surface display systems (Lpp_OmpA, Ag43 and INP) (Figure S2), except for the chimeric protein containing TEV protease in the Lpp_OmpA_SNAC(+) export system.

### 3.5. Effect of Export Systems on Growth of the Producing Strain

**Figure 4:**
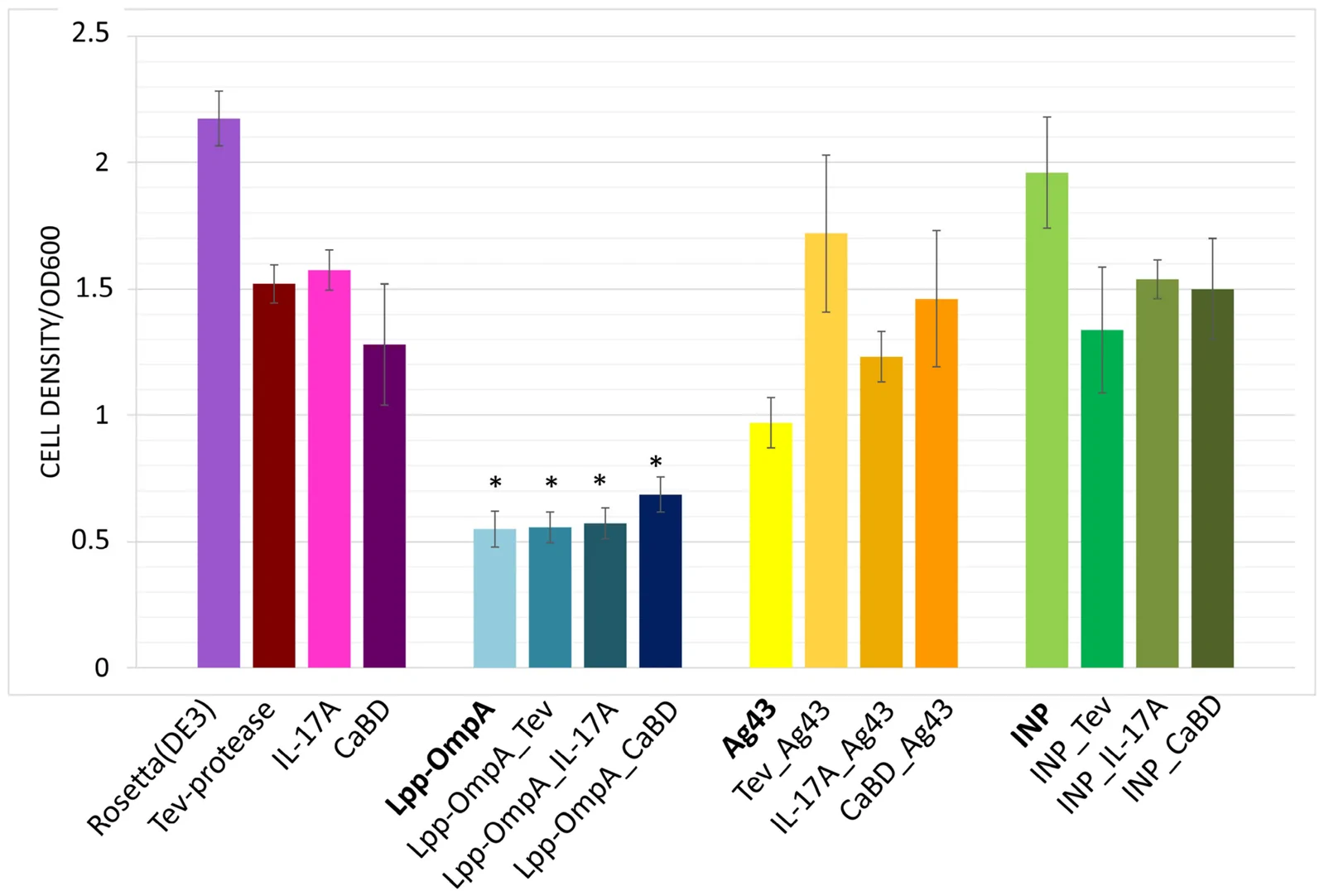
Comparison of the optical density (OD600) of *E. coli* cultures producing target proteins (TEV protease, IL-17A, CaBD) intracellularly and in three surface display systems (Lpp-OmpA_SNAC, SNAC_Ag43, INP_SNAC) three hours after inducer addition. The error bars, which indicate the standard deviation, were obtained from at least three independent experiments (three biological replicates). *—means *p*-value ≤ 0.05.

The production of membrane-anchoring motifs is well known to affect cell growth [6,27,50,51,52,53]. We compared optical densities of *E. coli* cell cultures 3 h after inducting gene expression from the chimeric constructs containing anchoring proteins from the three systems (Figure 4). Pairwise comparison was performed between two datasets—Rosetta(DE3) cells versus those overproducing different anchors, and cells overproducing cytoplasmic target proteins versus those with surface-displayed target proteins—to estimate the statistical significance of the obtained results. The *p*-value analysis showed that only the Lpp-OmpA anchor and its surface-displayed constructs with target proteins had a statistically significant effect on cell growth (Figure 4). One possible explanation is increased outer-membrane rigidity caused by excessive amounts of OmpA [54], which could impair cell division. Expression of the chimeric proteins in the Ag43 and INP export systems did not affect *E. coli* growth in such manner. Similar results were previously reported [6]: expression of a target protein in an Lpp-OmpA export system significantly slowed growth, whereas constructs based on the AIDA-I autotransporter and INP did not.

The overexpression of membrane protein genes can also overload the translocation system of *E. coli*, thus impeding the transport of essential endogenous membrane proteins required for vital cell functions [53,55,56].

### 3.6. Visualization of Target Protein Display on the Surface of E. coli Cells

Cell-surface display of the target proteins was confirmed using CaBD of nucleobindin 1 produced in two export systems. Fluorescent microscopy was used to localize CaBD in two recombinant protein display systems (Lpp_OmpA_SNAC and SNAC_Ag43) and in an intracellular expression system.

**Figure 5:**
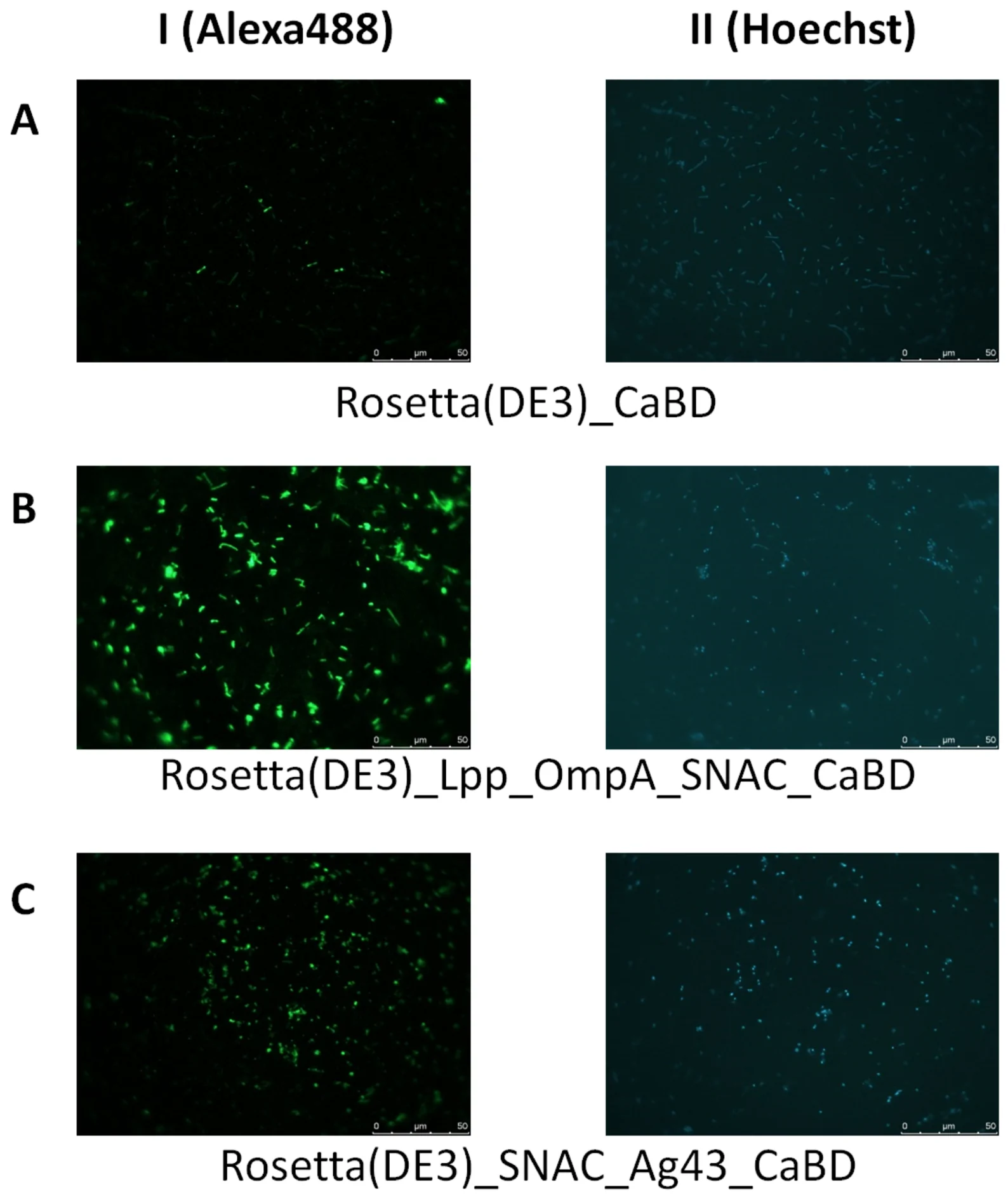
Fluorescent microscopy of CaBD expressed in the cytoplasm of *E. coli* Rosetta(DE3) cells (**AI**) or in the Lpp_OmpA_SNAC (**BI**) and SNAC_Ag43 (**CI**) cell-surface display systems. Cells immobilized on glass slides were first incubated with polyclonal antibodies against NUCB1 and then with secondary Alexa-488-conjugated anti-rabbit antibodies (green). DNA in *E. coli* cells was stained with Hoechst (blue) (**II**).

For this purpose, polyclonal serum against full-length NUCB1 and secondary anti-rabbit antibodies conjugated with Alexa488 were used [57]. A nucleic acid-binding Hoechst dye was used to locale the cells. As expected, CaBD was not detected on the surface of bacterial cells when it was not fused to a carrier protein, as indicated by the absence of fluorescence signal (Figure 5A). In the Lpp-OmpA_SNAC and SNAC_Ag43 export systems, almost all bacterial cells produced an Alexa488 fluorescent signal (Figure 5B,C), demonstrating the surface localization of the target protein.

None of the analyzed bacterial cultures, including those expressing only the Lpp-OmpA_SNAC or SNAC_Ag43 export platform constructs, showed nonspecific affinity for the Alexa-488-conjugated secondary anti-rabbit antibodies (Figure S3).

### 3.7. Production of Target Proteins in the Studied Export Systems

In this study, qualitative analysis of the possibility of production of functionally active target proteins using three export systems followed by chemical proteolysis was performed. Small volumes (50 mL) of cell culture were used. Recombinant protein production in the resulting export systems involves cleavage of the target protein from the bacterial cell surface by chemical proteolysis at a specific amino acid sequence, -G^SHHW- (the SNAC-tag) [43]. Unlike methods that use proteases to digest a specific site, this approach does not require an additional purification step to remove the cleavage agent. Ni^2+^ cleaves the -G^SHHW- sequence after the glycine residue. When the SNAC-tag is located before the N-terminus of the target protein, four amino acids are added to the protein; when it is located at the C-terminus, only a glycine residue remains attached to the target protein after cleavage. Data on protein production, cleavage efficiency, and protein recovery are presented in Supplementary Table S2.

#### 3.7.1. Production of Recombinant IL-17A and Assessment of Its Interaction with Specific Antibody

The target protein IL-17A was obtained by NiCl_2_-dependent chemical proteolysis from the cell surface of *E. coli* Rosetta(DE3) harboring the chimeric constructs. To optimize the cleavage conditions, incubation temperature and time were varied (18 °C, 25 °C, 37 °C; 1, 3, 18 h). The highest cleavage efficiency was observed at the maximal duration of incubation (18 h) at 37 °C (Supplementary Figure S4).

##### SNAC_Ag43 Export System

**Figure 6:**
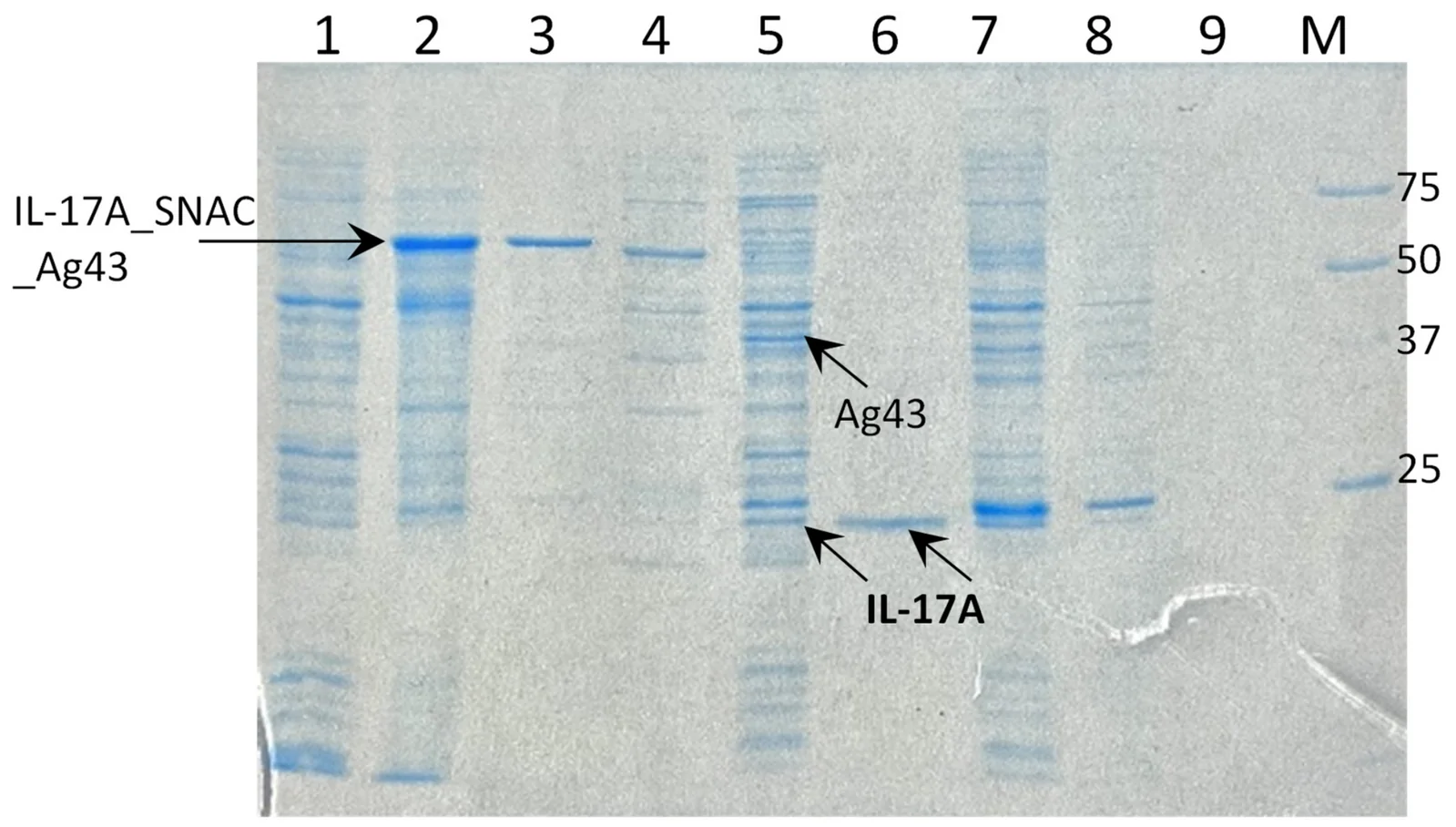
Production of IL-17A using the export system based on SNAC_Ag43 platform construct. 1—Rosetta(DE3)_IL-17A_SNAC_Ag43 cell pellet before the induction of chimeric IL-17A_SNAC_Ag43 construct expression; 2—Rosetta(DE3)_IL-17A_SNAC_Ag43 cell pellet 3 h after 0.5 mM of IPTG addition; 3–6—cells after expression of the chimeric IL-17A_SNAC_Ag43 construct; 3—cell pellet after incubation for 18 h at 37 °C without NiCl_2_; 4—supernatant without NiCl_2_, purified by Ni-NTA chromatography; 5—cell pellet after cleavage (incubation for 18 h at 37 °C in presence of NiCl_2_); 6—supernatant after NiCl_2_ cleavage, purified by Ni-NTA chromatography; 7—Rosetta(DE3)_IL-17A cell pellet producing intracellular IL-17A; 8—pellet of cells producing intracellular IL-17A; 9—supernatant of cells producing intracellular IL-17A after Ni-NTA chromatography; M—molecular weight marker (kDa).

When the export system based on SNAC_Ag43 platform construct was used, the chimeric protein IL-17A_SNAC_Ag43 was effectively cleaved into IL-17A and SNAC_Ag43 (Figure 6, lane 5; Supplementary Table S2). In the absence of NiCl_2_ under the same conditions (37 °C, 18 h), the chimeric protein IL-17A_SNAC_Ag43 was not cleaved (Figure 6, lane 3). After the cleavage, the part of IL-17A remained in the cell pellet (membrane fraction, Figure 6, lane 5), but the supernatant also contained the target protein. The supernatant was immobilized onto Ni-NTA Agarose and eluted with a buffer containing 250 mM of imidazole to obtain the final IL-17A preparation (Figure 6, lane 6). In the results, IL-17A recovery was 0.6 ± 0.3% (Supplementary Table S2). In the system without the autotransporter, a soluble form of IL-17A could not be obtained (Figure 6, lanes 7–9).

##### INP_SNAC Export System

**Figure 7:**
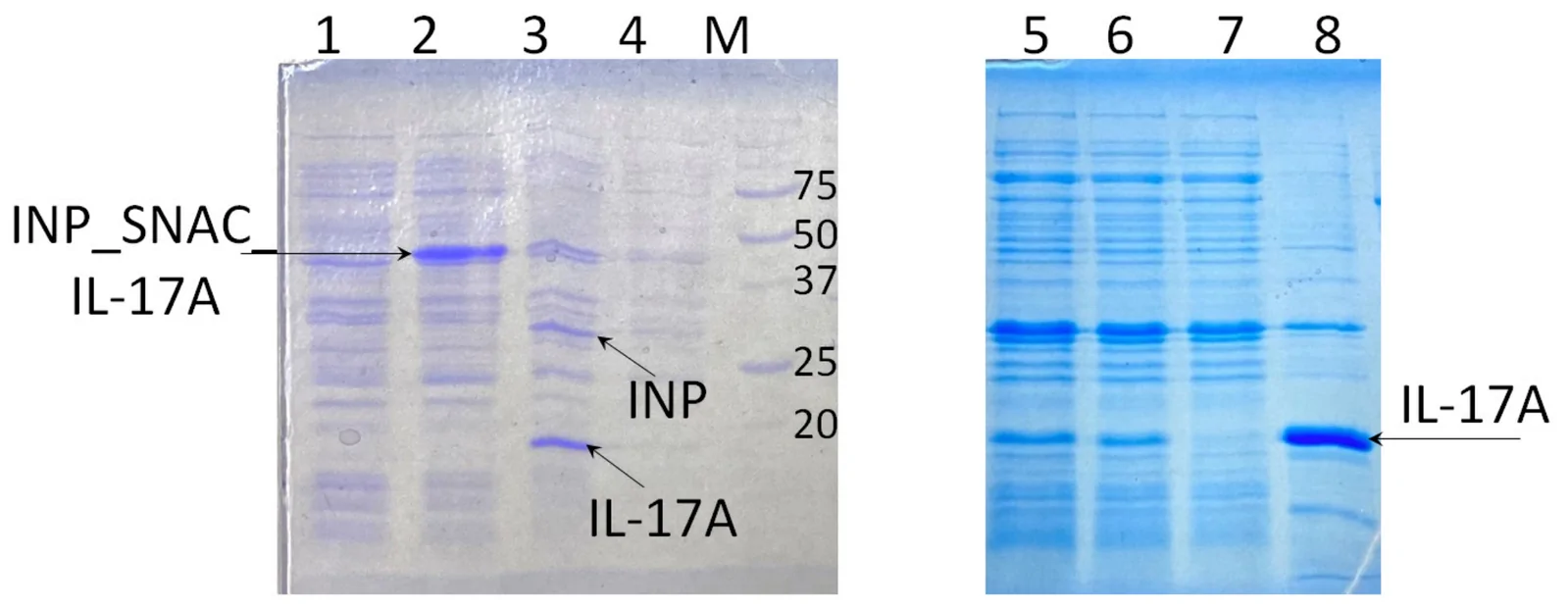
Preparation of IL-17A in INP_SNAC export system. 1—Rosetta(DE3)_INP_SNAC_IL-17A cell pellet before the expression of chimeric INP_SNAC_IL-17A construct; 2—Rosetta(DE3)_INP_SNAC_IL-17A cell pellet 3 h after addition of 0.5 mM of IPTG; 3, 4—cells expressing the chimeric INP_SNAC_IL-17A construct. 3—Cell pellet after cleavage with NiCl_2_. 4—Supernatant after cleavage with NiCl_2_; M—molecular weight marker (kDa). 5, 6—Cell pellet after cleavage with NiCl_2_ and subsequent urea treatment; 5—supernatant after incubation of cell pellet with 500 mM of urea; 6—supernatant after incubation of cell pellet with 1 M of urea; 7, 8—purification after treatment with 1 M of urea; 7—flow-through from Ni-NTA Agarose; 8—eluate from Ni-NTA Agarose.

In this export system, the protein remained associated with the cell membrane after the cleavage of IL-17A and was therefore present in the cell pellet (Figure 7, lanes 3, 4). To destabilize the noncovalent interactions between the target protein and the cell membrane, the pellet was treated after chemical proteolysis with 1% of Triton, 500 mM of Gu, 1 M of NaCl, and 500 mM or 1 M of urea. Treatment with urea allowed IL-17A to be obtained in soluble form (Figure 7, lanes 5, 6). This preparation was immobilized onto Ni-NTA Agarose and eluted with a buffer containing 250 mM of imidazole, resulting in a pure IL-17A preparation (Figure 7, lane 8).

##### Lpp_OmpA_SNAC Export System

**Figure 8:**
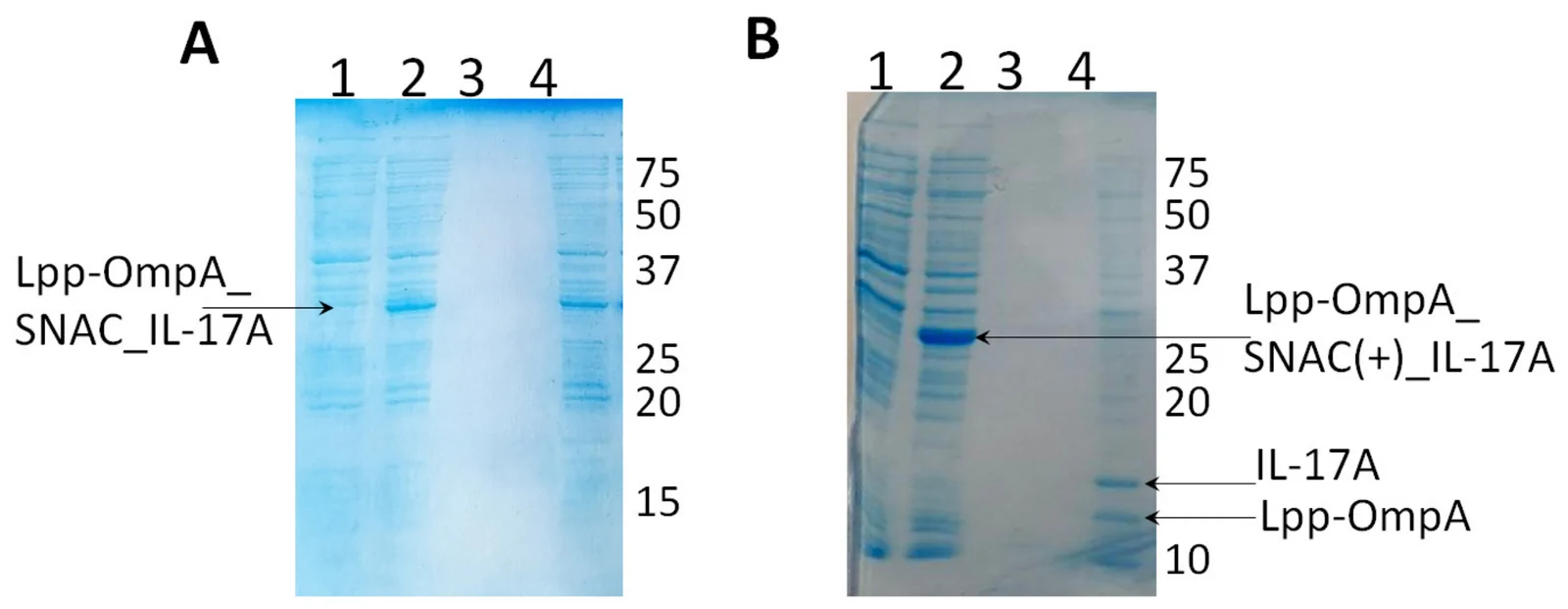
Production of IL-17A in Lpp-OmpA export system. (**A**) Production of IL-17A in Lpp-OmpA_SNAC system. 1—Rosetta(DE3)_Lpp-OmpA_SNAC_IL-17A cell pellet before the induction of Lpp-OmpA_SNAC_IL-17A construct expression; 2—Rosetta(DE3)_Lpp-OmpA_SNAC_IL-17A cell pellet 3 h after addition of 0.5 mM of IPTG; 3, 4—cells expressing chimeric construct Lpp-OmpA_SNAC_IL-17A: 3—supernatant after NiCl_2_ cleavage; 4—cell pellet after NiCl_2_ cleavage. (**B**) Production of IL-17A in Lpp-OmpA_SNAC(+) system. 1—Rosetta(DE3)_Lpp-OmpA_SNAC(+)_IL-17A cell pellet before the induction of Lpp-OmpA_SNAC(+)_IL-17A construct expression; 2—Rosetta(DE3)_Lpp-OmpA_SNAC(+)_IL-17A cell pellet 3 h after addition of 0.5 mM of IPTG; 3, 4—cells expressing chimeric Lpp-OmpA_SNAC(+)_IL-17A construct; 3—supernatant after cleavage with NiCl_2_; 4—cell pellet after cleavage with NiCl_2_.

In the export system based on Lpp-OmpA_SNAC platform, the target protein was not cleaved from the cell surface in presence of NiCl_2_; the chimeric protein Lpp-OmpA_SNAC_IL-17A remained in the cell pellet (Figure 8A, lane 4). To assess the efficiency of the chemical proteolysis of -G^SHHW- sequence in this construct, we disrupted *E. coli* Lpp_OmpA_SNAC_IL-17A cells under denaturing conditions and purified the chimeric protein using Ni-NTA Agarose in presence of 2 M of urea. Dang et al. [43] showed that 2 M of urea does not affect the chemical cleavage of -G^SHHW- sequence. However, urea did not facilitate cleavage of the target protein in the Lpp_OmpA_SNAC export system.

We hypothesized that the linker in this system was too short for effective cleavage of the target protein. A construct with modified chemical cleavage site and elongated linker between the anchoring protein and the cleavage site was created (Lpp_OmpA_SNAC(+)_IL-17A) (Figure 1B). A short G^SHHW sequence is sufficient for effective nickel-ion-mediated chemical cleavage in most cases, whereas other cases require the longer YFLPG^SHHWG sequence [43]. This extended sequence of SNAC enabled NiCl_2_-mediated chemical proteolysis of the chimeric protein in this export system (Figure 8B).

The chimeric Lpp_OmpA_SNAC(+)_IL-17A protein was cleaved, but the resulting IL-17A protein remained in the pellet (Figure 8B, lane 4; Supplementary Table S2). Treatment with various denaturing agents (Section 3.7.1) did not yield soluble IL-17A; in this export system, the protein remained associated with the membrane (Figure S5).

##### Analysis of the Specific Interaction of IL-17A with Antibody

To analyze the resulting IL-17A preparations produced using INP_SNAC and SNAC_Ag43 export systems, the interaction of IL-17A with its known binding partner, the netakimab antibody (BIOCAD, Russia), was examined [47]. Ribosomal protein L1, which does not interact with interleukin, was used as a negative control [58,59]. As shown above, IL-17A produced using SNAC_Ag43 and INP_SNAC export systems possesses 6His-sequence, whereas both the antibody and L1 protein lack this sequence. Thus, retention of a protein partner on Ni-NTA Agarose containing immobilized IL-17A indicates formation of a protein–protein complex.

**Figure 9:**
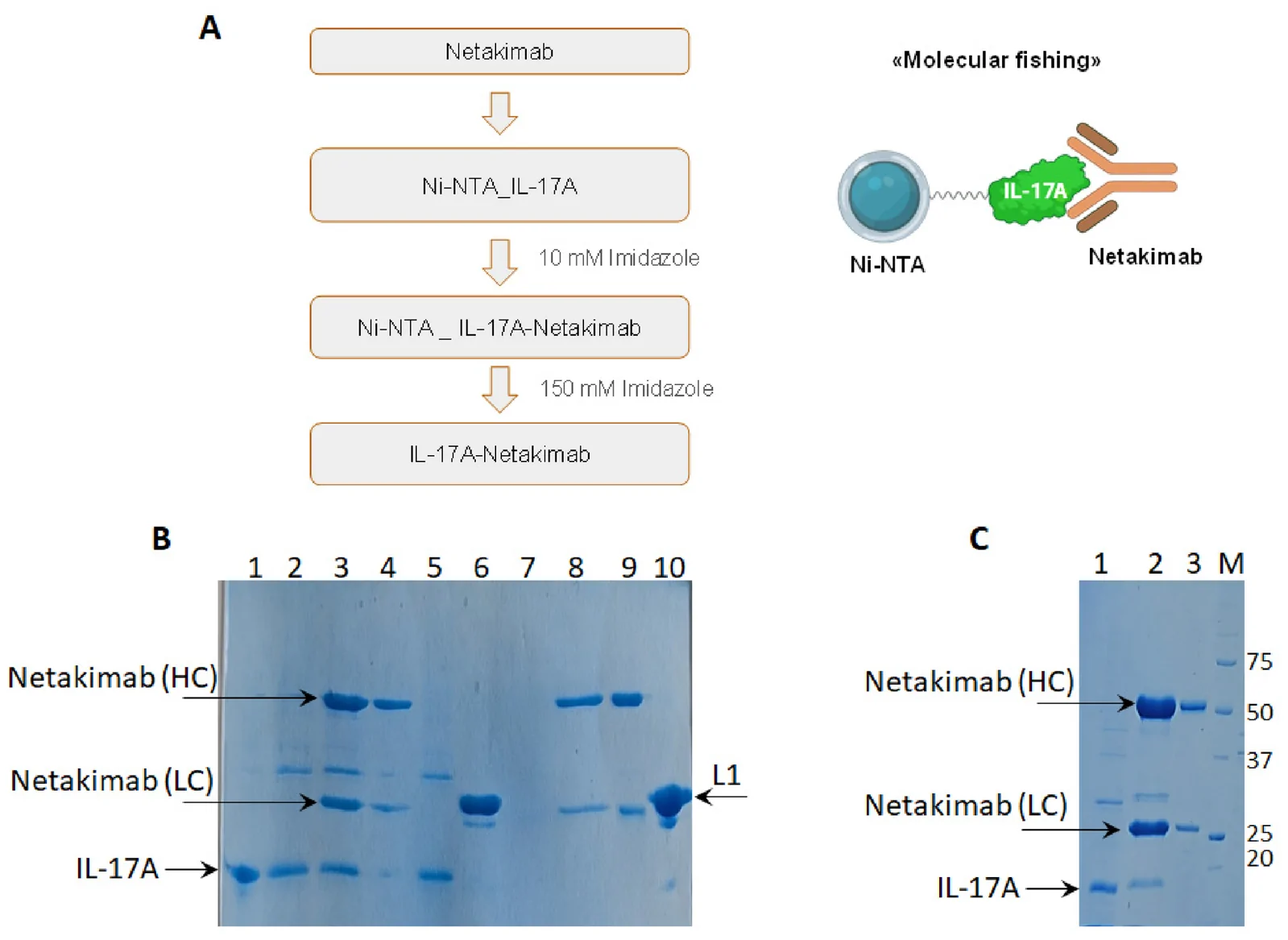
Analysis of interaction of IL-17A and netakimab antibody. (**A**). Design of analysis of netakimab interaction with IL-17A preparation immobilized onto Ni-NTA Agarose. (**B**). Electrophoretic analysis of interaction of IL-17A produced in INP_SNAC system with netakimab on Ni-NTA Agarose. 1—IL-17A, control; 2, 5—IL-17A immobilized on the resin; 3—eluate of IL-17A:netakimab complex; 4—flow-through after application of netakimab to the resin with immobilized IL-17A; 6—flow-through after application of ribosomal protein L1; 7—eluate after application of netakimab onto the resin without IL-17A and washing; 8—flow-through after application of netakimab onto the resin without IL-17A; 9—netakimab, control; 10—ribosomal protein L1, control. (**C**). Electrophoretic analysis of interaction of IL-17A produced in SNAC_Ag43 system with netakimab on Ni-NTA Agarose. 1—IL-17A immobilized on the resin; 2—eluate of IL-17A:netakimab complex; 3—netakimab, control; M—molecular weight markers (kDa).

After netakimab was applied to Ni-NTA Agarose containing immobilized IL-17A at a 1.2:1 molar ratio, no netakimab was detected in the flow-through, whereas the eluate (250 mM of imidazole) contained both IL-17A and netakimab, demonstrating formation of an IL-17A–netakimab complex (Figure 9B, lane 3; Figure 9C, lane 2). The free antibody passed through Ni-NTA Agarose in the absence of IL-17A (Figure 9B, lane 8), and ribosomal protein L1 was also present in the flow-through after being applied to resin containing immobilized IL-17A (Figure 9, lane 6).

Thus, IL-17A produced using the SNAC_Ag43 and INP_SNAC export systems was confirmed to interact with netakimab.

#### 3.7.2. Catalytic Domain of TEV Protease: Isolation and Activity Assessment

The soluble preparation of active TEV protease was obtained only using INP_SNAC export system.

##### INP_SNAC

**Figure 10:**
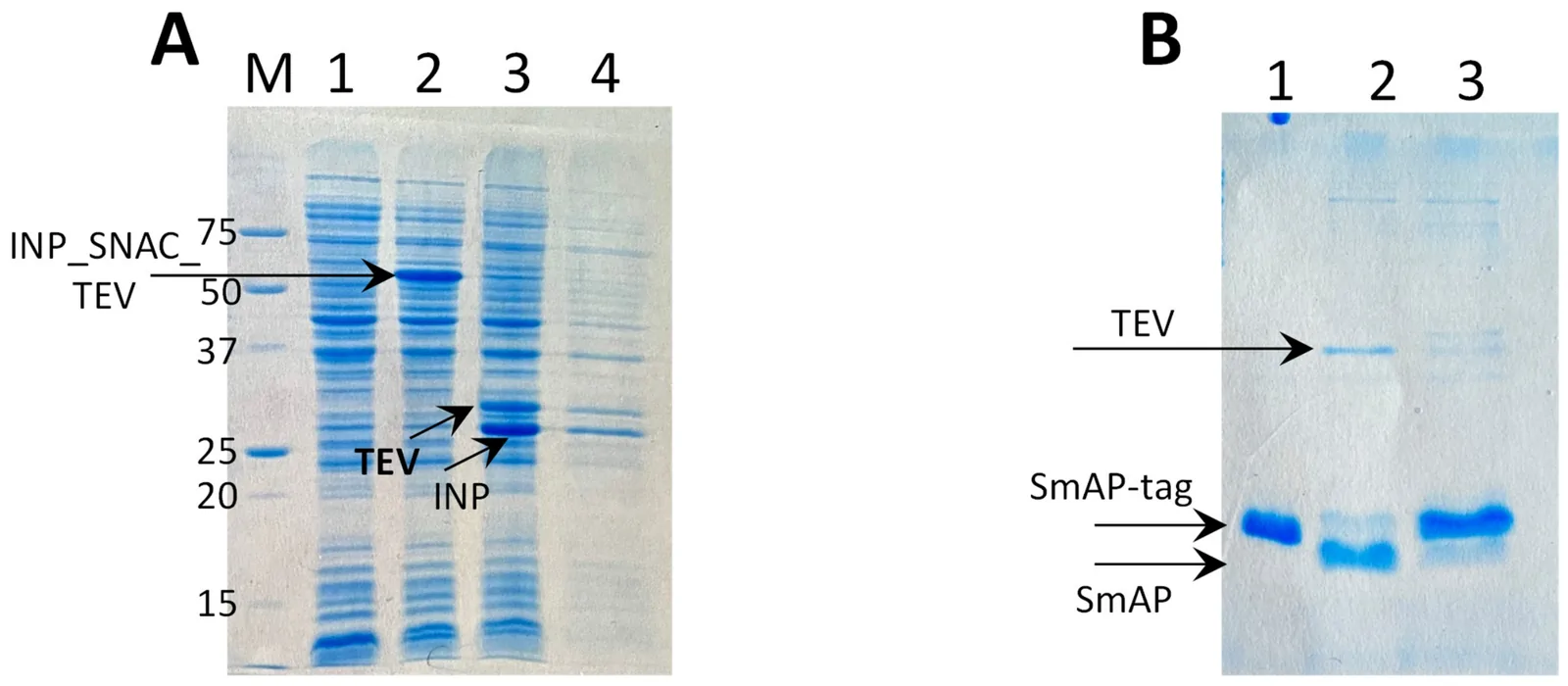
Production of TEV protease in three export systems and analysis of proteolytic activity of the preparations. (**A**). Production of TEV protease in INP_SNAC export system. M, molecular weight marker (kDa); 1—Rosetta(DE3)_INP_SNAC_TEV cell pellet before the induction of chimeric INP_SNAC_TEV construct expression; 2—Rosetta(DE3)_INP_SNAC_TEV cell pellet 3 h after addition of 0.5 mM of IPTG; 3 and 4—cells with expression of chimeric INP_SNAC_TEV construct; 3—cell pellet after NiCl_2_ cleavage; 4—supernatant after NiCl_2_ cleavage and Ni-NTA Agarose chromatography. (**B**). Activity analysis of TEV protease produced in INP_SNAC export system: 1—SmAP2-6xHis-tag, untreated control; 2—SmAP2-6xHis-tag after proteolysis by recombinant cytoplasmic TEV protease; 3—SmAP2-6xHis-tag after proteolysis by TEV protease obtained in INP_SNAC system.

After chemical proteolysis of chimeric protein INP_SNAC_TEV protease, the soluble fraction contained a small amount of TEV protease. Ni-NTA Agarose chromatography was performed for further purification of this protein (Figure 10A).

##### SNAC_Ag43

In the SNAC_Ag43 export system, TEV protease remained associated with the cell membrane after chemical proteolysis. Treatment with 2 M of urea did not release the protein into the soluble fraction (Figure S6A).

##### Lpp_OmpA_SNAC

The chimeric protein Lpp-OmpA_SNAC_TEV was not cleaved, as was also observed for IL-17A (Figure S6B). The level of TEV protease production in the modified Lpp_OmpA_SNAC(+)_TEV export system was very low, precluding further studies with this construct.

##### Analysis of TEV Protease Activity

As shown above, the functional preparation of TEV protease was obtained only with the INP_SNAC export system.

The activity of TEV protease was assessed by proteolysis of an archaeal protein from the SmAP2 family possessing TEV protease cleavage site [60]. The activity of TEV protease obtained using the export system was compared with the activity of TEV protease produced using cytoplasmic expression system without recombinant protein surface display.

Figure 10B demonstrates that TEV protease obtained using the cell-surface export system successfully cleaved the substrate protein. The band intensities of both the cleaved and non-cleaved substrates on the electrophoregram were quantified using ImageJ software. The average values and maximum deviations are provided for a single biological replicate. The efficiency of the TEV protease produced by the INP_SNAC export system was 12 ± 5%, compared to 83 ± 8% for the control recombinant TEV protease. Based on the standard definition of TEV protease activity (where one unit cleaves 2 μg of hybrid protein), the average activity of the purified preparations was estimated as follows: 8000 units/mg for the cytoplasmic TEV protease and 1200 units/mg for the TEV protease obtained from the INP_SNAC export system.

#### 3.7.3. Preparation of Recombinant CaBD of Nucleobindin 1 Using Export System

**Figure 11:**
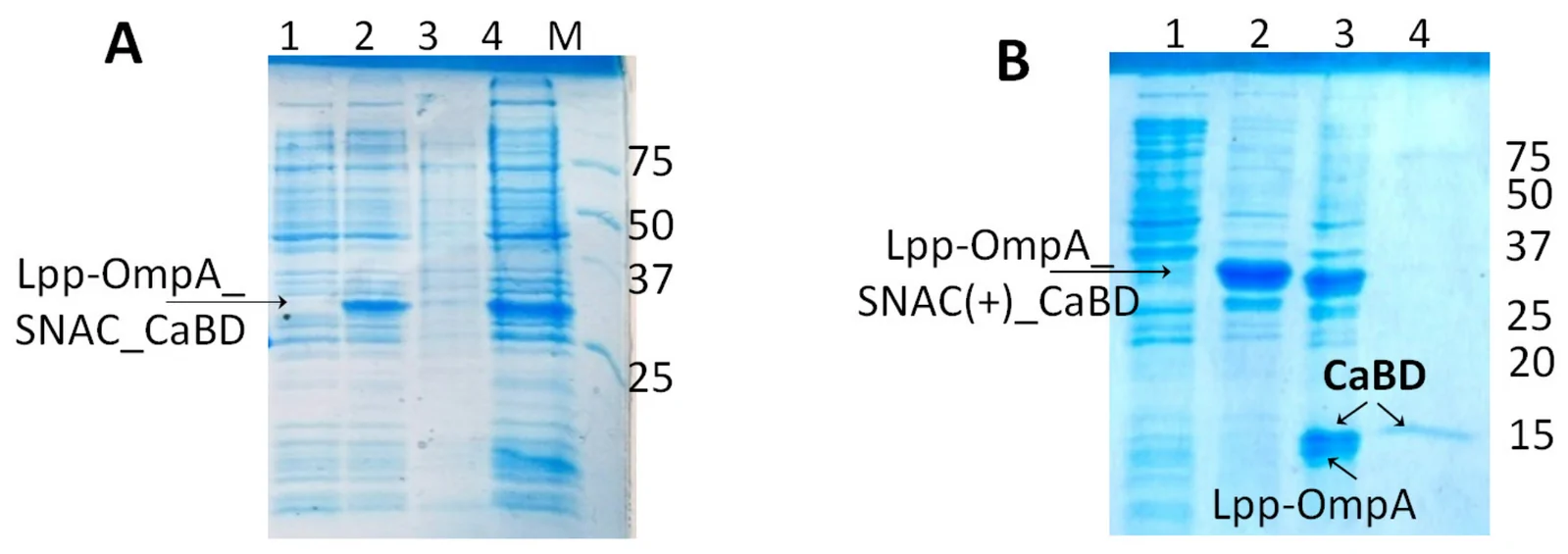
CaBD production using the Lpp_OmpA_SNAC and Lpp_OmpA_SNAC(+) export systems. (**A**). CaBD production in export system based on Lpp-OmpA_SNAC platform. (1) Rosetta(DE3)_Lpp-OmpA_SNAC_CaBD cell pellet before induction of chimeric Lpp-OmpA_SNAC_CaBD construct expression; (2) Rosetta(DE3)_Lpp-OmpA_SNAC_CaBD cell pellet 3 h after addition of 0.5 mM of IPTG; (3) supernatant after cleavage by NiCl_2_ purified by Ni-NTA chromatography; (4) cell pellet after cleavage by NiCl_2_; M—molecular weight marker (kDa). (**B**). CaBD production in export system based on Lpp-OmpA_SNAC(+) platform. (1) Rosetta(DE3)_Lpp-OmpA_SNAC(+)_CaBD cell pellet before induction of chimeric Lpp-OmpA_SNAC_CaBD construct expression; (2) Rosetta(DE3)_Lpp-OmpA_SNAC(+)_CaBD cell pellet 3 h after addition of 0.5 mM of IPTG; (3) cell pellet after cleavage by NiCl_2_; (4) supernatant after cleavage by NiCl_2_ purified by Ni-NTA Agarose chromatography.

CaBD was produced using the *E. coli* cell-surface display systems and the same procedures used for IL-17A and TEV protease (Figure 11 and Figure S7).

As observed for IL-17A and TEV protease, CaBD was not cleaved from the *E. coli* cell surface in the system based on the Lpp_OmpA_SNAC platform construct and remained in the cell pellet. However, in the modified Lpp_OmpA_SNAC(+)_system, CaBD was efficiently cleaved from the cell surface, and a fraction of the protein was recovered in soluble form (Figure 11).

##### Analysis of the Specific Interaction of CaBD with RNA Fragment

**Figure 12:**
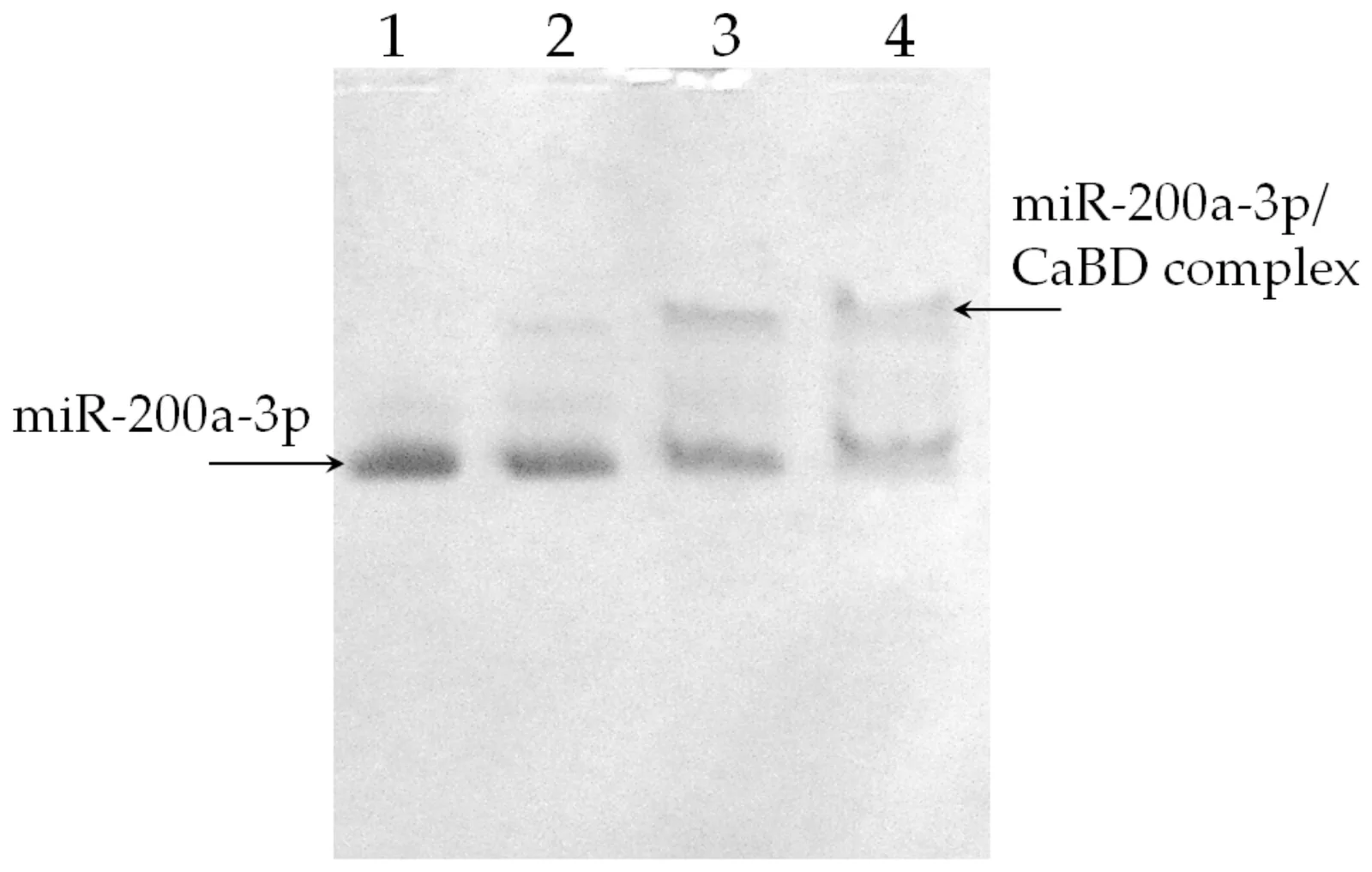
Analysis of the specific interaction of CaBD with RNA fragment. (1) miR-200a-3p; (2) miR-200a-3p complexed with CaBDc (RNA:protein molar ratio, 1:0.5); (3) miR-200a-3p complexed with CaBDc (RNA:protein molar ratio, 1:1); (4) miR-200a-3p complexed with CaBD produced using the Lpp_OmpA_SNAC(+) export system (RNA:protein molar ratio, 1:1).

Recently, we demonstrated the RNA-binding activity of CaBD [46]. To test this activity, an electrophoretic mobility shift assay (EMSA) was used (Figure 12), along with employment of the same biotinylated, chemically synthesized RNA oligomer (miR-200a-3p) described in [46]. The results showed that CaBD produced using the Lpp_OmpA_SNAC(+) export system can form a complex with an RNA containing a specific binding site, similar to the CaBD produced in the cytoplasmic expression system (CaBDc).

## 4. Discussion

Our study covered three surface display expression systems, three model proteins exported by these systems, one *E. coli* strain, and a limited set of IPTG-induction conditions. The goals and methods of our study differ from approaches used to create whole-cell biocatalysts, which are employed mainly in laboratory diagnostics or bioremediation. We combined established systems for exposing target proteins to the cell surface with site-directed chemical proteolysis. The proteins’ recovery was ≤1.4% in four out of the twelve target-protein/expression system combinations and 0% in the other eight variants (Supplementary Table S2). However, despite the low recovery, this approach holds promise for producing recombinant proteins. This work presents preliminary data and hypotheses about possible relationships between export architecture and target-protein properties. These hypotheses require further refinement and verification.

**Hypothesis** **1.** *Hypothetical considerations regarding the effect of recombinant protein display systems on protein production.*

The Lpp_OmpA export system displayed the model proteins; however, the cleavage site became accessible only after the linker was extended. We hypothesized that this may be related to the small size of OmpA (approximately 45 × 20 Å) relative to the Ag43 and INP (approximately 60 × 25 Å). Molecule dimensions were measured using PyMol viewer. The peptidoglycan layer in Gram-negative bacteria can be up to 60 Å [61]; therefore, the association of target proteins with the membrane in these systems might result from nonspecific interactions with neighboring proteins or lipid molecules. CaBD from NUCB1 was the only exception which was effectively produced using this system after linker extension. It should also be noted that this export system caused a significant decrease in the strain’s growth rate. Apparently, an excessive amount of OmpA in the cell membrane may increase membrane rigidity and slow cell division.

IL-17A was produced using AT SNAC_Ag43 export system in soluble form, whereas TEV protease remained associated with the membrane (Supplementary Table S2).

The INP_SNAC export platform enabled both IL-17A and TEV protease to be obtained in soluble form. The chimeric proteins were cleaved at the cell surface, and a fraction of TEV proteases appeared in the soluble fraction. However, IL-17A could be isolated from the membrane fraction only after treatment with a buffer solution supplied with 1 M of urea and subsequent dialysis against urea-free buffer solution. A method for releasing SNAC-tagged GFP immobilized on the cell surface was described in 2019 [43]. The authors suggested that this method does not require additional purification steps. However, another study evaluated a recombinant protein purification platform based on peptidoglycan particles bound to SNAC-tagged GFP molecules [62] and showed that a substantial amount of GFP remained associated with the peptidoglycan particles after nickel-dependent cleavage. The addition of 0.05% SDS doubled the protein yield. In our study, treatment with 1 M of urea enabled IL-17A to be isolated in a soluble form and to be accessible for interaction with specific antibodies. A 1 M urea solution is generally known not to impair protein structure [63,64,65]. The activity of the resulting IL-17A preparation was demonstrated by its specific interaction with netakimab.

**Hypothesis** **2.** *Hypothetical considerations regarding the effect of the structural properties of displayed recombinant protein on their isolation procedure.*

Proteins with different properties were used as targets for heterologous expression. The human IL-17A homodimer consists of two 17 kDa monomers, each comprising two antiparallel β-sheets (PDB ID: 4HR9). The catalytic domain of viral TEV protease forms a β-structure with a single C-terminal α-helix (PDB ID: 1LVM). The calcium-binding domain of human nucleobindin 1 (12 kDa) possesses calcium-binding EF-hand motifs and comprises four α-helixes and a short β-sheet (6 aa) (PDB ID: 1SNL). The CaBD structure determined by NMR demonstrates high conformation mobility of the protein. Moreover, a hydrophobic surface of CaBD becomes exposed in calcium-bound form, which may facilitate protein–protein interactions [66].

Soluble CaBD was obtained only using the Lpp_OmpA export system. In this case, some molecules are likely to be in the calcium-free form and therefore possess a more hydrophilic surface, weakening their interaction with other proteins or lipids.

TEV protease obtained using the Lpp_OmpA_SNAC and SNAC_Ag43 export systems remained in the pellet after cleavage. Amphipathic helices are known to bind membrane lipids [67]. Since the cleavage site was close to the protein’s C-terminal helix, the C-terminal helix may have interacted with the cell membrane after cleavage, causing the enzyme to remain in the cell pellet. The low activity of TEV protease obtained using INP_SNAC export system may have resulted from incorrect folding of the C-terminal α-helix because its amino acid residues participate in formation of the TEV protease catalytic center.

Homodimeric IL-17A was obtained using two export systems—the SNAC_Ag43 and INP_SNAC. Apparently, since IL-17A consists mainly of β-structural elements, its association with the cell membrane is weaker than that of proteins containing α-helical structures.

## 5. Conclusions

This study presents basic laboratory-scale research aimed at a comparative qualitative assessment of bacterial surface display expression systems for the production of different recombinant proteins.

This work represents the initial stage in the search for an optimal surface display expression system for producing recombinant proteins. At this stage, the assessment of the system’s efficiency was primarily qualitative rather than quantitative.

Analysis of *E. coli* cell growth demonstrated that the Lpp-OmpA export system had an inhibitory effect, in contrast to the Ag43 autotransporter system and the system based on P. syringae INP. Moreover, use of the Lpp-OmpA system requires particular attention to the linker sequence and cleavage site.

Usage of SNAC_Ag43 and INP_SNAC export systems allows one to produce recombinant proteins in soluble form, with its recovery of up to 1.4%. At the same time, successful protein recovery requires individual optimization.

## Data Availability

The original contributions presented in this study are included in the article/Supplementary Materials. Further inquiries can be directed to the corresponding author.

## Supplementary Materials

The following supporting information can be downloaded at https://www.mdpi.com/article/10.3390/biotech15040078/s1, Figure S1: Analysis of expression of anchoring constructs in three surface-display systems; Figure S2: Target proteins expression in three surface display systems; Figure S3: Control experiments with fluorescence microscopy; Figure S4: Optimization of cleavage conditions for the TEV_SNAC_Ag43 and INP_SNAC-IL-17A chimeric proteins using NiCl_2_; Figure S5: Production of recombinant IL-17A using the Lpp-OmpA_SNAC(+) export system; Figure S6: Production of TEV protease using the SNAC_Ag43 and Lpp-OmpA_SNAC export systems; Figure S7: Production of CaBD using the SNAC_Ag43 and INP_SNAC export systems; Table S1: Primers used in this study; Table S2: Protein production level, cleavage efficiency and protein recovery for three target proteins obtained using Lpp_OmpA_SNAC, Lpp_OmpA_SNAC(+), SNAC_Ag43, and INP_SNAC export systems.
