# Supplementary material for: Engineering of Surface Display Systems in E. coli for Recombinant Protein Production
Source: BioTech (Basel). 2026 Sep 15;15(4):78. doi: 10.3390/biotech15040078 (PMC13600108; doi:10.3390/biotech15040078)
Supplement: Supplementary file 1 [file biotech-15-00078-s001.zip › biotech-4531258-supplementary.pdf]

Engineering of surface display systems in *E. coli* for recombinant protein production

Olga S. Kostareva, Polina I. Blinova, Svetlana V. Tishchenko, Ilya A. Kolyadenko, Anatoly S. Glukhov, Irina A. Eliseeva and Alisa O. Mikhaylina *

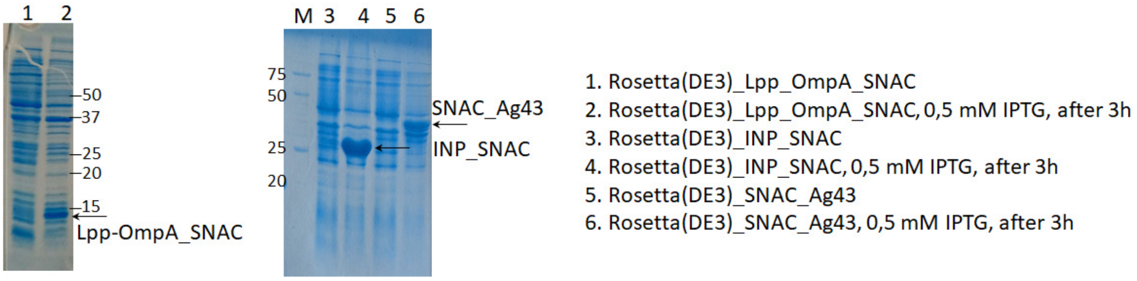

Figure S1. Analysis of expression of anchoring constructs in three surface-display systems.

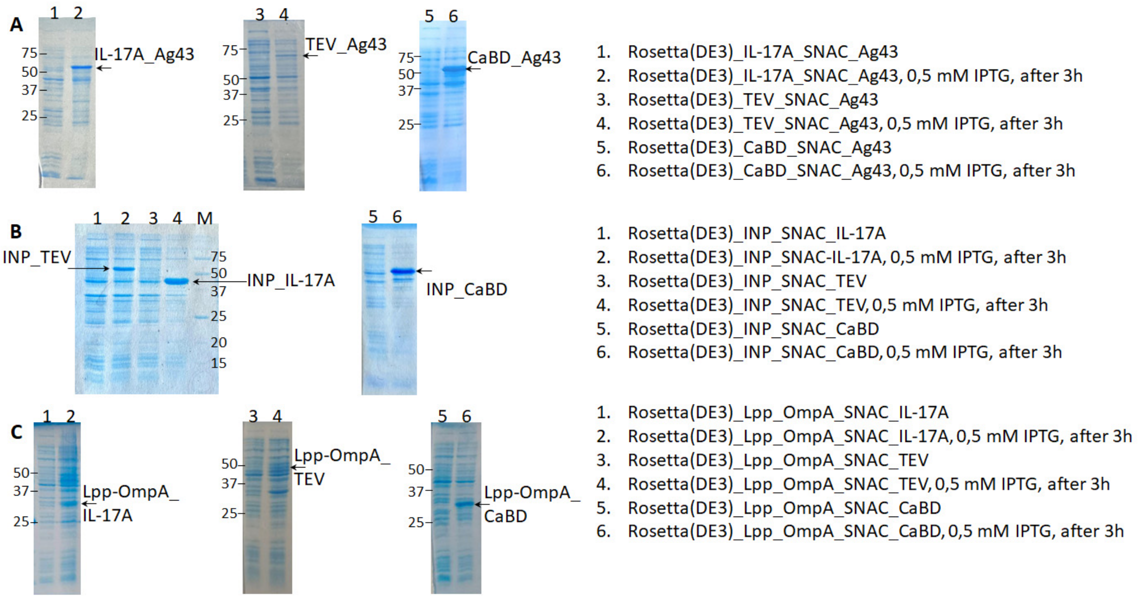

Figure S2. Target proteins expression in three surface display systems. A) Expression of target proteins hybrid constructions using the SNAC_Ag43 export system. B) Expression of target proteins hybrid constructions using the INP_SNAC export system. C) Expression of target proteins hybrid constructions using the Lpp_OmpA_SNAC export system.

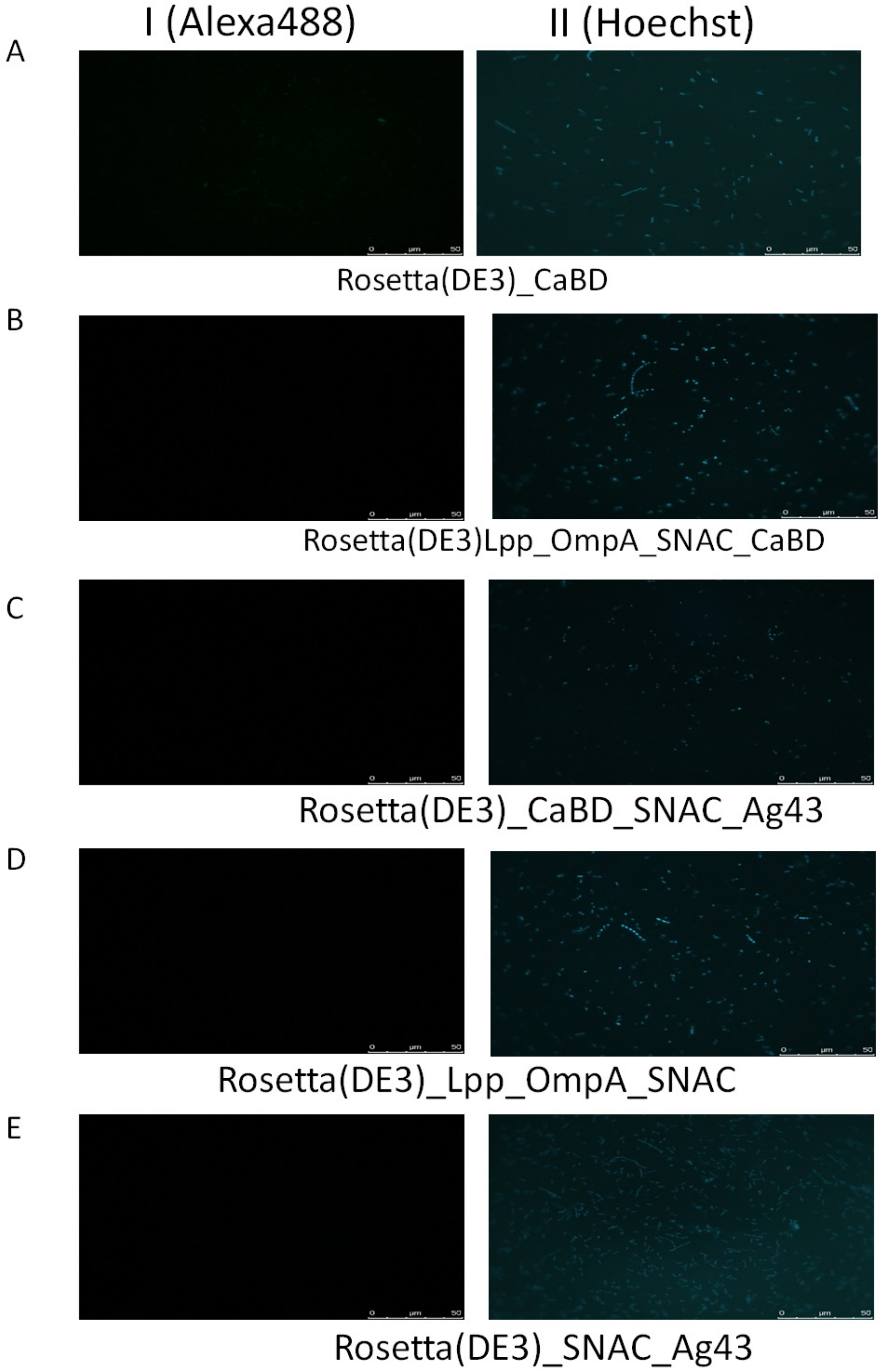

Figure S3. Control experiments with fluorescence microscopy. CaBD expressed in *E. coli* Rosetta(DE3) cells in the cytoplasm (Ai) and as a part of export systems to the cell surface Lpp_OmpA_SNAC (B I) and SNAC_Ag43 (C I); and membrane anchors OmpA (D I) and Ag43 (E I) expressed in *E. coli* Rosetta(DE3) cells. Cells immobilized on glass were incubated with a secondary Alexa-488-conjugated anti-rabbit antibody. DNA in *E. coli* cells is stained with Hoechst (blue) (II).

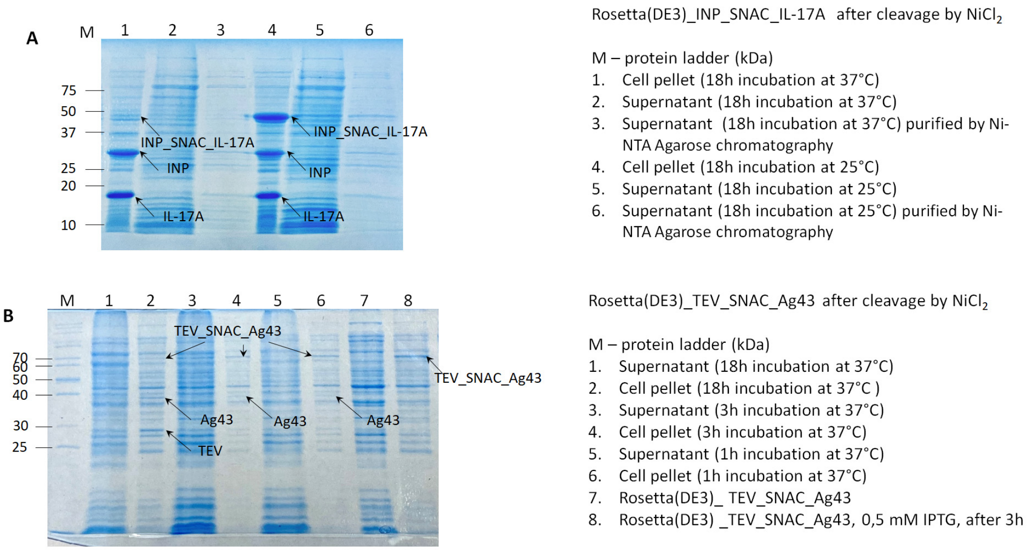

Figure S4. Optimization of cleavage conditions for the TEV_SNAC_Ag43 and INP_SNAC-IL-17A chimeric proteins using $NiCl_2$. A. Optimization of cleavage conditions for the INP_SNAC-IL-17A chimeric protein. Lanes: 1) cell pellet after 18h incubation at 37°C; 2) supernatant after 18h incubation at 37°C; 3) supernatant (18h incubation at 37°C) after cleavage by $NiCl_2$ purified by Ni-NTA Agarose chromatography; 4) cell pellet after 18h incubation at 25°C; 5) supernatant after 18h incubation at 25°C; 6) supernatant (18h incubation at 25°C) after cleavage by $NiCl_2$ purified by Ni-NTA Agarose chromatography. B. Optimization of cleavage conditions for the TEV_SNAC_Ag43 chimeric protein. Lanes: M – molecular weight marker (kDa); 1) supernatant after 18h incubation at 37°C; 2) cell pellet after 18h incubation at 37°C; 3) supernatant after 3h incubation at 37°C; 4) cell pellet after 3h incubation at 37°C; 5) supernatant after 1h incubation at 37°C; 6) cell pellet after 1h incubation at 37°C; 7) Rosetta(DE3)_TEV_SNAC_Ag43 cell pellet before induction of chimeric TEV_SNAC_Ag43 construct; 8) Rosetta(DE3)_TEV_SNAC_Ag43 cell pellet 3 hours after addition 0.5 mM IPTG.

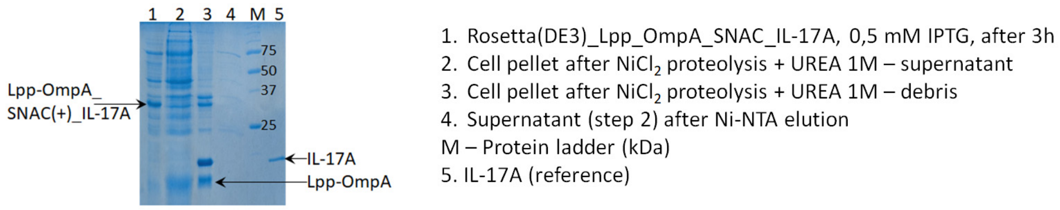

Figure S5. Production of recombinant IL-17A using the Lpp-OmpA_SNAC(+) export system. 1) cell pellet after expression of the chimeric Lpp-OmpA_SNAC(+)_IL-17A; 2)-4) cell pellet after cleavage (incubation for 18 h at 37 ^{0}C in presence of $NiCl_2$): 2) supernatant after incubation of cell pellet with 1M urea; 3) cell pellet after incubation of cell pellet with 1M urea; 4) supernatant after incubation of cell pellet with 1M urea purified by Ni-NTA Agarose chromatography; M –molecular weight marker (kDa); 5) IL-17A (reference).

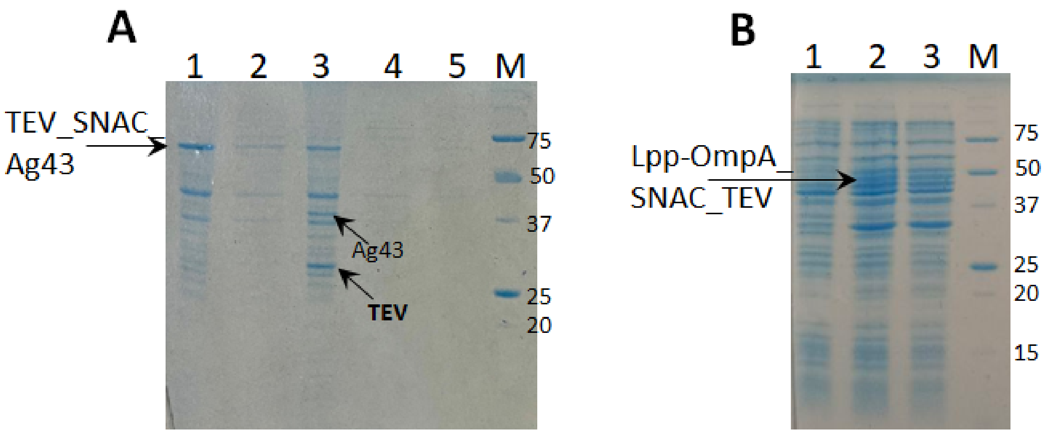

Figure S6. Production of TEV protease using the SNAC_Ag43 and Lpp-OmpA_SNAC export systems. A. Production of TEV protease using the SNAC_Ag43 export system. 1) cells expressing the chimeric TEV_SNAC_Ag43; 2) Supernatant after cleavage with $NiCl_2$; 3) Cell pellet after cleavage with $NiCl_2$; 4)-5) Cell pellet after cleavage with $NiCl_2$: 4) supernatant after incubation of cell pellet with 1M urea; 5) supernatant after incubation of cell pellet with 2M urea; M –molecular weight marker (kDa). B. Production of TEV protease using the Lpp-OmpA_SNAC export system. 1) Rosetta(DE3)_Lpp-OmpA_SNAC_TEV cell pellet before the expression of chimeric Lpp-OmpA_SNAC_TEV construct; 2) Rosetta(DE3)_Lpp-OmpA_SNAC_TEV cell pellet 3 hours after addition of 0.5 mM IPTG; 3) Cell pellet after cleavage with $NiCl_2$; M – molecular weight marker (kDa).

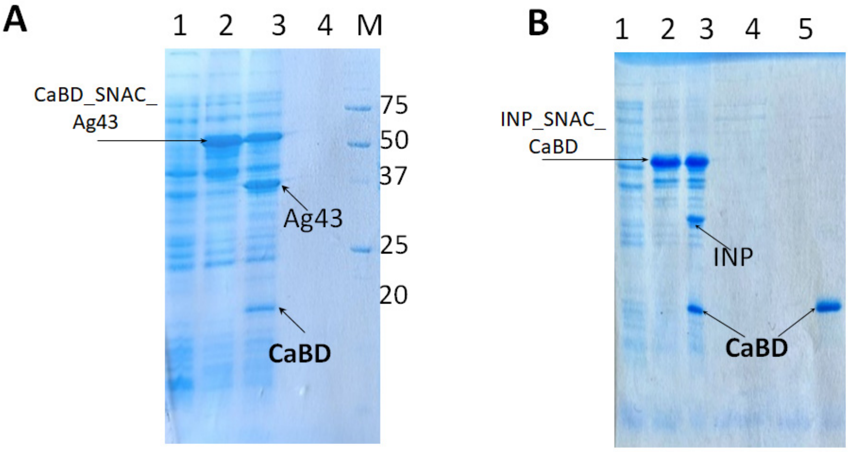

Figure S7. Production of CaBD using the SNAC_Ag43 and INP_SNAC export systems. A. CaBD production using the SNAC_Ag43 export system. Lanes: 1) Rosetta(DE3)_CaBD_SNAC_Ag43 cell pellet before induction of chimeric CaBD_SNAC_Ag43 construct expression; 2) Rosetta(DE3)_CaBD_SNAC_Ag43 cell pellet 3 hours after addition of 0.5 mM IPTG; 3) cell pellet after cleavage by $NiCl_2$; 4) supernatant after cleavage by $NiCl_2$ purified by Ni-NTA Agarose chromatography; M – molecular weight marker (kDa). B. CaBD production using the INP_SNAC export system. 1) Rosetta(DE3)_INP_SNAC_CaBD cell pellet before induction of chimeric INP_SNAC_CaBD construct; 2) Rosetta(DE3)_INP_SNAC_CaBD cell pellet 3 hours after addition 0.5 mM IPTG; 3) supernatant after cleavage by $NiCl_2$; 4) cell pellet after cleavage by $NiCl_2$; 5) supernatant after cleavage by $NiCl_2$ purified by Ni-NTA Agarose chromatography; 6) CaBD (reference).

Table S1. Primers used in this study. F – forward primer; R – reversed primer; The nucleotide sequence encoding and complementary to the SNAC-tag region, is highlighted in brown-green; The nucleotide sequence encoding and complementary to Lpp sequence is highlighted in blue; The nucleotide sequence encoding and complementary to OmpA sequence is highlighted in turquoise; The nucleotide sequence encoding and complementary to the His-tag sequence is highlighted in gray; The nucleotide sequences complementary to the vectors sequence (pET-11a, pET-22b(+)) are underlined; The nucleotide sequence encoding and complementary to the SP of PelB is highlighted in yellow; The nucleotide sequence encoding the linker is highlighted in italics; The extended nucleotide sequence encoding SNAC-tag sequence is boxed.

	Name	Sequence, 5'→3'
P1	F Lpp	ATGAAAGCTACTAAACTGGTACTGG
P2	R Lpp-OmpA	CAAAGCCAACATACGGGTTAATTCCTTGATCGATTTTAGCGTTGCTG-<br>GAGC
P3	F OmpA-Lpp	CTAAAATCGATCAaGGAATTAACCCGTATGTTGGCTTTG
P4	R OmpA_SNAC	CCAATGATGAGATCCGTTGTCTGGACGAGTACCTATGGTG
P5	F pet11a	TAAGGATCCCGGGTACCTCGAGTCG
P6	R pet11a	ATGTATATCCTCCTATTCTTAAAGTTAAACAAAATTATTTC
P7	R SNAC-pet11	CGACTCGAGGTACCCGGGATCCTTACCAATGATGAGATCCGTTGTCC
P8	F Lpp-pet11a	CTTTAAGAATAGGAGGATATACATATGAAAGCTACTAAACTGGTAC
P9	F TEV	GGTCATCATCATCATCATCATCACGGC
P10	R TEV	ACGACGGCGGCGACGGTTCATCAG
P11	F Il-17A	CATCATCATCATCATCACAGCAGCGGC
P12	R Il-17A-	AGCCACATGGTGTACGATTGGAGTCAC
P13	F CaBD	CATTGGCATCATCATCATCATCACAGCAGC
P14	R CaBD	TTATCAAAACTCCTTCCTCTGAGTGG
P15	F IL-17a-SNAC	GTCCAGACAACGGATCTCATCATTGGCATCATCATCATCATCACAG-<br>CAGCGGCC
P16	R IL-17a-pet11	CGACTCGAGGTACCCGGGATCCTTAAGCCACATGGTGTAC-<br>GATTGGAGTCACGC
P17	F Tev-pet11	GTCCAGACAACGGATCTCATCATT-<br>GGGGTCATCATCATCATCATCATCACGGCG
P18	R Tev-pet11	CGACTCGAGGTACCCGGGATCCTTAACGACGGCGGCGAC-<br>GGTTCATCAGC
P19	F CaBD-pet11	GTCCAGACAACGGATCTCATCATTGGCATCATCATCATCATCAC
P20	R CaBD-pet11	CGACTCGAGGTACCCGGGATCCTTATCAAAACTCCTTCCTCTGAG-<br>TGG
P21	F Omp+linker+snac	*GAAGCGGAGGAGGAAGC*TATTTTCTGCCGGGCTCTCATCATT-<br>GGGGTCATCATCATCATCATCAT
P22	R Omp+linker+snac	AAATA*GCTTCCTCCTCCGCTTCCTCCTCC*GTTGTCTGGACGAG-<br>TGCCGATG

	Name	Sequence, 5'→3'
P23	F PelB	ATGAAATACCTGCTGCCGACCGCTGCTGCTGGTCTGCTGCTCCTT-GCTGCTC
P24	R PelB	CCAATGATGAGATCCTGCCATCGCCGGTTGAGCAGCAAGGAGCAG-CAGACC
P25	F Ag43	GGATCTCATCATTGGCTGCGCAGTGAAAATGCTTATCGTGC
P26	R Ag43	TTAGAAGGTCACATTCAGTGTGGCCTGACCGTTATACCC
P27	For Ag43 pET11	CTTTAAGAATAGGAGGATATACATATGAAATACCTGCTGCCGACC
P28	R Ag43 pET11	CGACTCGAGGTACCCGGGATCCTTAGAAGGTTACATTCAGTGTGGCC
P29	R pET Ag43	TGCCATAGCCGGTTGAGCAGC
P30	F petAg43 TEV	CCTTGCTGCTCAACCGGCGATGG-CAGGTCATCATCATCATCATCATCAC
P31	R petAg43 Tev	CACTGCGCAGCCAATGATGAGATCCACGACGGCGGCGACGGTTC
P32	F pETAg IL-17A	CCTTGCTGCTCAACCGGCGATGGCACATCATCACATCATCACAG
P33	R pETAg IL-17A	CACTGCGCAGCCAATGATGAGATCCAGCCACATGGTGTACGATTGG
P34	F pETAg CaBD	CCTTGCTGCTCAACCGGCGATGGCACATCATCATCATCATCACAG-TAGCG
P35	R pETAg CaBD	CACTGCGCAGCCAATGATGAGATCCAAACTCCTTCCTCTGAG-TCGATGCG
P36	F INP	ATGAATCTCGACAAGGCGTTGGTGCTG
P37	R INP	CCAGTGGTGGCTTCC*TCCGCTTCCTCCTCC*AATCAGATCACTGTGGTT-GCCAGCAGTCTCG
P38	R pet22b	GGCCATCGCCGGTTGTGCAGCTAAGAGCAGCAGACC
P39	F pet22b	CACCACCACCACCACCACTGAGATC
P40	F pet22-INP	CTTAGCTGCACAACCGGCGATGGCCATGAATCTCGACAAGGCGTT-GGTGCTG
P41	R SNAC-pet22	GATCTCAGTGGTGGTGGTGGTGGTGCCAG-TGGTGGCTTCC*TCCGCTTCCTCCTCC*
P42	F pet22b_INP	TGAGATCCGGCTGCTAACAAATCCCG
P43	R pet22b_INP	GTGGTGGTGGTGGTGGTGCCAGTGG
P44	F Tev-INP	CACCACTGGCACCACCACCACCACCACGGCGAAAGCTT-GTTCAAAGGTC
P45	R Tev-pet22	CGGGATTTGTTAGCAGCCGGATCTCAACGACGACGACGAC-GGTTCATCAGC
P46	F IL-17a-INP	CACCACTGGCATCACCACCACCACCACAGCAGCGGCCTGGTTCCTCG
P47	R IL-17a-pet22	CGGGATTTGTTAGCAGCCGGATCTCAAGCCACATGGTGTAC-GATTGGAGTCACGC
P48	F CaBD-INP	CACCACTGGCACCACCACCACCACCACAGCAGCGGCCTGGT
P49	R CaBD-pet22	CGGGATTTGTTAGCAGCCGGATCTCAAAACTCCTTCCTCTGAGTGG

The nucleotide sequence of export systems genetic constructs and its translation:

Lpp_OmpA_SNAC

Lpp SP+1st 9aa, OmpA mature 46-159 aa, SNAC

ATGAAAGCTACTAAACTGGTACTGGGCGCGGTAATCCTGGGTTCTACTCTGCTGGCAGGTT-
GCTCCAGCAACGCTAAAATCGATCAGGGAATTAACCCGTATGTTGGCTTTGAAATGGGTTACGAC
TGGTTAGGTCGTATGCCGTACAAAGGCAGCGTTGAAAACGGTGCATACAAA-
GCTCAGGGCGTTCAACTGACCGCTAAACTGGGTTACCCAATCACTGACGACCTGGACATCTACAC
TCGTCTGGGTGGCATGGTATGGCGTGCAGACACTAAATCCAACGTTTATGGTAAAAACCAC-
GACACCGGCGTTTCTCCGGTCTTCGCTGGCGGTGTTGAGTACGCGATCACTCCTGAAATCGCTAC
CCGTCTGGAATACCAGTGGACCAACAACATCGGTGACGCACACACCATCGGCACTCGTCCG-
GACAACGGCTCTCATCATTGG

MKATKLVLGAVILGSTLLAGCSSNAKIDQGINPYVGFEMGYDWLGRMPYKGSVENGA-
YKAQGVQLTAKLGYPITDDLDIYTRLGGMVWRADTKSNVYGKNHDTGVSPVFAGGVEYAITPEIATRL
EYQWTNNIGDAHTIGTRPDNGSHHW

Lpp_OmpA_SNAC(+)

Lpp SP+1st 9aa, OmpA mature 46-159 aa, *linker*, SNAC extra SNAC

ATGAAAGCTACTAAACTGGTACTGGGCGCGGTAATCCTGGGTTCTACTCTGCTGGCAGGTT-
GCTCCAGCAACGCTAAAATCGATCAGGGAATTAACCCGTATGTTGGCTTTGAAATGGGTTACGAC
TGGTTAGGTCGTATGCCGTACAAAGGCAGCGTTGAAAACGGTGCATACAAA-
GCTCAGGGCGTTCAACTGACCGCTAAACTGGGTTACCCAATCACTGACGACCTGGACATCTACAC
TCGTCTGGGTGGCATGGTATGGCGTGCAGACACTAAATCCAACGTTTATGGTAAAAACCAC-
GACACCGGCGTTTCTCCGGTCTTCGCTGGCGGTGTTGAGTACGCGATCACTCCTGAAATCGCTAC
CCGTCTGGAATACCAGTGGACCAACAACATCGGTGACGCACACACCATCGGCACTCGTCCG-
GACAAC*GGAGGAGGAAGGGAGGAGGAAGC*TATTTTCTGCCGGGCTCTCATCATTGG

MKATKLVLGAVILGSTLLAGCSSNAKIDQGINPYVGFEMGYDWLGRMPYKGSVENGA-
YKAQGVQLTAKLGYPITDDLDIYTRLGGMVWRADTKSNVYGKNHDTGVSPVFAGGVEYAITPEIATRL
EYQWTNNIGDAHTIGTRPDNGGGSGGGSYFLPGSHHW

SNAC_Ag43

SPpelB_SNAC_Ag43, 700-1039aa

ATGAAATACCTGCTGCCGAC-
CGCTGCTGCTGGTCTGCTGCTCCTCGCTGCCCAGCCGGCGATGGCCGGCTCTCATCATTGGCTGCG
CAGTGAAAATGCTTATCGTGCAGAAGTCCCCCTG-
TATGCCTCCATGCTGACACAGGCAATGGACTATGACCGGATTGTGGCAGGCTCCCGCAGCCATC
AGACCGGTGTAAATGGTGAAAACAACAGCGTCCGTCTCAG-
CATTCAGGGCGGTCATCTCGGTCACGATAACAATGGCGGTATTGCCCGTGGGGCCACGCCGGAA
AGCAGCGGCAGCTATGGATTCGTCCGTCTGGAGGGTGACCTGATGAGAACAGAGGTTGCCGG-
TATGTCTGTGACCGCGGGGGTATATGGTGCTGCTGGCCATTCTTCCGTTGATGTTAAGGATGATGA
CGGCTCCCGTGCCGGCACGGTCCGGGATGATGCCGGCAGCCTGGGCGGATACCTGAATCTGG-
TACACACGTCCTCCGGCCTGTGGGCTGACATTGTGGCACAGGGAACCCGCCACAGCATGAAAGC
GTCATCGGACAATAACGACTTCCGCGCCCGGGGCTGGGGCTGGCTGGGCTCACTGGAAAC-
CGGTCTGCCCTTCAGTATCACTGACAACCTGATGCTGGAGCCACAACTGCAGTATACCTGGCAGG

GACTTTCCCTGGATGACGGTAAGGACAACGCCGGTTATGTGAAGTTCGGGCATGGCAGTG-
CACAACATGTGCGTGCCGGTTTCCGTCTGGGCAGCCACAACGATATGACCTTTGGCGAAGGCACC
TCATCCCGTGCCCCCCTGCGTGACAGTGCAAAACACAGTGTGAGTGAATTAC-
CGGTGAACTGGTGGGTACAGCCTTCTGTTATCCGCACCTTCAGCTCCCGGGGAGATATGCGTGTG
GGGACTTCCACTGCAGGCAGCGGGATGACGTTCTCTCCCTCACAGAATGGCACATCACTG-
GACCTGCAGGCCGGACTGGAAGCCCGTGTCCGGGAAAATATCACCCTGGGCGTTCAGGCCGGTT
ATGCCCACAGCGTCAGCGGCAGCAGCGCTGAAGGGTATAACGGTCAGGCCACAC-
TGAATGTGACCTTCTAA

MKYLLPTAAAGLLLLAAQPAMAGSHHWLRSE-
NAYRAEVPLYASMLTQAMDYDRIVAGSRSHQTGVNGENNSVRLSIQGGHLGHDNNGGIARGATPESS
GSYGFVRLEGDLMRTEVAGMSVTAGVYGAAGHSSVDVKDDDGSRAGTVRDDAGSLG-
GYLNLVHTSSGLWADIVAQGTRHSMKASSDNNDFRARGWGWLGSLETGLPFSITDNLMLEPQLQYT
WQGLSLDDGKDNAGYVKFGHGSAQHVRAGFRLGSHNDMTFGEGTSSRAPLRD-
SAKHSVSELPVNWWVQPSVIRTFSSRGDMRVGTSTAGSGMTFSPSQNGTSLDLQAGLEARVRENITLGV
QAGYAHSVSGSSAEGYNGQATLNVTF

INP_SNAC:
INPN 1-167 aa*, *INPCRD 168-203 aa*, *linker* *SNAC

ATGAATCTCGACAAGGCGTTGGTGCTGCGTACCTGTGCAAATAACATGGCCGATCATT-
GCGGCCTTATATGGCCCGCTTCTGGCACGGTGGAATCCAAATACTGGCAGTCAACCAGGCGGCA
TGAGAATGGTCTGGTCGGTTTACTGTGGGGCGCTGGAACCAGCGCTTTTCTAAGCGTG-
CATGCCGATGCGCGATGGATTGTCTGTGAAGTCGCCGTTGCCGACATCATCAGTCTGGAAGAACC
GGGAATGGTCAAGTTTCCGCGGGCCGAGGTGGTTCATGTCGGCGACAG-
GATCAGCGCGTCACACTTTATTTCGGCACGTCAGGCCGACCCTGCATCAACGCCAACGCCAATG
ACCGCGGCCACGCCCCCACCCACGCCCGCGACAGCAAATGTCACGTTAC-
CGGTGGCCGAACAGGCCAGTCATGAAGTGTTCGATGTGGCGTTGGTCAGCGCGGCTGCCCCCCC
GGTAAATACCCTGCCGGTGACGACGCCGCAGAATTTGCAGACCGCCACTTACGGCAGCAC-
GTTGAGCGGCGACAACCACAGTCGACTCATTGCCGGTTATGGCAGTAACGAGACTGCTGGCAAC
CACAGTGATCTGATTGGAGGAGGAAGCGGAGGAAGCCACCACTGG

MNLDKALVLRTCANNMADHCGLIWPASGTVESKYWQSTRRHENGLVGLLWGAG-
TSAFLSVHADARWIVCEVAVADIISLEEPGMVKFPRAEVVHVGDRISASHFISARQADPASTPTPM
TAATPPPTPATANVTLPVAEQASHEVFDVALVSAAAPPVNTLPVTTPQNLQTATYGSTLSGDNHSR
LIAGYGSNETAGNHSDLIgggsgGSHHW

Table S2. Protein production level, cleavage efficiency and protein recovery for three target proteins obtained using Lpp_OmpA_SNAC, Lpp_OmpA_SNAC(+), SNAC_Ag43, and INP_SNAC export systems. The parameters have been estimated by gel densitometry following SDS-PAGE using Image J software. Concentrations of final preparations for all target protein have been estimated spectrophotometrically. Cleavage efficiency has been calculated as the sum of cleavage target protein and anchor. Means and maximum deviations for all target proteins across three biological replicates are presented in the table.

Target protein	Protein production, mg/L	Cleavage efficiency, %	Protein recovery,%
**Lpp_OmpA_SNAC**			
IL-17A	34,5±13,8	0	0
TEV protease	9,5±8,0	0	0
CaBD	64,4±9,3	0	0
**Lpp_OmpA_SNAC(+)**			
IL-17A	73,6±11,8	86±8	0
TEV protease	0	0	0
CaBD	103,5±21,6	61±13	0,24±0,1
**SNAC_Ag43**			
IL-17A	69±15	76±10	0,6±0,3
TEV protease	18,0 ±4,5	62±10	0
CaBD	69±11	60±12	0
**INP_SNAC**			
IL-17A	71±12	88±7	1,4±0,03
TEV protease	92±20	82±8	0,27±0,1
CaBD	66,7±9,2	42±18	0
